# Promoting Functional Mobility in Individuals with Non-Ambulatory Cerebral Palsy: A Scoping Review of the MOVE Programme

**DOI:** 10.3390/children13020292

**Published:** 2026-02-20

**Authors:** Riclef Schomerus, Ginny S. Paleg, Roslyn W. Livingstone, Britta Dawal, Liane Bächler

**Affiliations:** 1 Research Unit ‘Participation in Impaired Physical and Motor Development’, Department of Rehabilitation Sciences, TU Dortmund University, 44227 Dortmund, Germany; riclef.schomerus@tu-dortmund.de (R.S.); liane.baechler@tu-dortmund.de (L.B.); 2 CanChild, McMaster University, Hamilton, ON L8S 1C7, Canada; 3 School of Health Sciences, University of KwaZulu-Natal, Private Bag X54001, Durban 4000, South Africa; roslyn.livingstone@ubc.ca; 4 Occupational Science and Occupational Therapy, Faculty of Medicine, University of British Columbia, Vancouver, BC V6T 2B5, Canada; 5 Department of Educational and Social Sciences, University of Applied Sciences Southwestfalia, 59494 Soest, Germany; dawal.britta@fh-swf.de

**Keywords:** cerebral palsy, non-ambulatory, functional mobility, locomotion, MOVE programme, intervention, rehabilitation

## Abstract

**Highlights:**

**What are the main findings?**

- MOVE has been implemented for almost 40 years in a wide variety of clinical and educational settings to enhance functional mobility with non-ambulatory individuals.
- There is currently more descriptive rather than experimental evidence published in relation to the MOVE programme.

**What are the implications of the main findings?**

- MOVE can be considered an activity-based approach that may be used to facilitate family-centred goals and enhance participation in meaningful activities in line with contemporary theories and clinical guidelines.
- Further experimental research is required to elucidate the programme’s mechanisms and further establish its effectiveness.

**Abstract:**

Background/Objective: Mobility Opportunities Via Education (MOVE) is a structured intervention to enhance independent mobility skills in individuals who are non-ambulatory. This study aims at identifying and mapping the literature related to the MOVE programme and to describe its content according to preselected categories, focusing on individuals with non-ambulatory cerebral palsy. Methods: A scoping review was conducted, with thirteen databases searched in May 2024, complemented by reference search and private databases; the search was updated in August 2025. Publications after 1985 were included without restrictions on language, population, or context. Two reviewers independently screened records and extracted data using qualitative content analysis. Results: From 6794 records, 228 publications in 15 languages were included, mainly from the United States and Europe. MOVE was developed in the 1980s during a shift towards age-appropriate, functional interventions for individuals with severe disabilities. It is an early task-specific, activity-based and family-centred approach with retrospectively proposed foundations in dynamic systems theory and motor learning. Implementation follows a structured six-step process, embedding mobility training into daily routines. MOVE has been implemented across populations, settings, and countries, particularly for non-ambulatory individuals with cerebral palsy.

## 1. Introduction

The prevalence of cerebral palsy (CP) ranges from 1.6 per 1000 live births in high-income countries (HICs) to approximately 3.4 per 1000 in low- and middle-income countries (LMICs) [1]. The locomotor function of individuals with CP is commonly categorised using the expanded and revised Gross Motor Function Classification System (GMFCS) [2]. In HICs, approximately 20% to 30% of children and adolescents with CP function at GMFCS Levels IV and V, indicating substantial or complete dependence on others. In LMICs, this proportion is markedly higher, ranging from 48% in Brazil [3] to 59% in Indonesia [4].

These individuals experience significant challenges in performing everyday activities and typically have limited opportunities for participation. Physical activity levels are consistently low, while sedentary behaviour is high [5,6], increasing the risk of long-term health conditions such as cardiometabolic diseases, as well as a shortened lifespan [7].

Interventions aimed at enhancing motor function are recognised as core components of the often complex and multidisciplinary care programmes [8]. Current clinical guidelines recommend that such interventions focus on functional, meaningful goals identified by the individual and their caregivers, and be delivered in natural environments [9]. To yield meaningful outcomes, they typically require high levels of intensity and consistency [9,10]. As these conditions are often difficult to achieve in routine therapy settings, structured home programmes are recommended as additional components in the treatment schedules [11]. While these programmes may be acknowledged by parents, they also can contribute to caregiver burden, which is already high in primary caregivers of non-ambulatory children and adolescents, and interfere with parental roles [12].

Children and adolescents spend a large portion of their waking time in childcare settings, schools, and day centres. These environments may therefore offer opportunities to integrate motor learning into daily routines and substantially increase intervention dosage. To date, this potential appears to be underutilised, since individuals who are non-ambulatory are sedentary most of their time in educational and residential settings [13].

Beyond clinical outcomes, the broader implementation of the MOVE programme must be considered within the framework of healthcare planning. Individuals with severe disabilities often require well-coordinated, long-term healthcare and social support [14]. By identifying where and in which populations the MOVE programme has been applied, this review will provide a necessary evidence base to inform planning and resource allocation, and to identify possible underused settings. Mapping these diverse contexts is essential to ensure that rehabilitation services meet the actual needs of the target population across all stages of life.

One programme specifically developed to address this potential is Mobility Opportunities Via Education (MOVE). Introduced in 1990, MOVE promotes goal-oriented, functional motor learning, integrated into the daily schedules within natural settings such as classrooms and residential homes. Despite its practical relevance, the programme has received relatively limited attention in rehabilitation research and practice to date.

A comprehensive overview of the literature published about the MOVE programme is currently lacking. A broader understanding of the programme may expand treatment options for individuals with non-ambulatory cerebral palsy, as well as other severe motor impairments, possibly leading to enhanced long-term outcomes for these populations. It may provide additional resources for practitioners and highlight opportunities for interdisciplinary collaboration across the boundaries of medical, therapeutic, and educational professions. Finally, it may contribute to the evidence base available for policymakers.

The purpose of this scoping review is to systematically map and describe the existing literature on MOVE.

The overarching review questions are:

- What are the literature characteristics regarding publication year, language, geographic location, publication type, and relationship to MOVE?
- How is MOVE characterised, including history and development, concept, conceptual precursors and theoretical foundations, role of assessments and equipment?
- What recommendations and material for implementation exist?
- How has MOVE been adapted across various populations and settings?

Article results are intended to serve as a starting point for future research into evidence-based implementation of the MOVE programme.

## 2. Materials and Methods

This review was conducted according to the JBI methodology for scoping reviews [15], and is reported following the Preferred Reporting Items for Systematic reviews and Meta Analysis extension for Scoping Reviews (PRISMA-ScR) Guideline [16]. A protocol was registered on the Open Science Framework on 1 February 2024 (https://osf.io/zseb5/?view_only=fac554e7c92643439cec6c9e99e42d1e, accessed on 1 February 2024).

Articles related to the MOVE programme published after 1985 were included in this review, as 1986 marked the beginning of programme development. To enable a comprehensive identification of all the literature available on the MOVE programme, no restrictions were set on population or outcome. Furthermore, no limits were set on the language, article type or place of publication. Articles were excluded if they were subjective or promotional in nature.

In March 2024, a pilot search was conducted on MEDLINE and ERIC via EBSCOhost to refine the search strategy. Between 8 and 16 May 2024, the following research platforms were officially searched: ten scientific databases (Cinahl, ERIC, PSYNDEX, Education source, Pubmed MEDLINE, APA PsychInfo, APA PsychArticles and Academic Search Ultimate—all via EBSCOhost—plus SCOPUS and ProQuest), three library databases (BASE, LIVIVO, and WorldCat), and the search engine Google Scholar. The search was updated on 25 August 2025. Additional sources were identified by contacting MOVE-related organisations (MOVE International, MOVE Europe, MOVE Austria and Germany, Rifton Equipment), screening reference lists, and consulting authors’ personal databases, which comprised non-indexed theses and articles. These additional publications were assessed for eligibility by consensus of the reviewers. Supplementary Table S1 provides an overview of all databases searched, the number of results retrieved, and any relevant search restrictions.

The search strategy combined broader terms (e.g., locomotor rehabilitation, non-ambulatory, gait trainer) with MOVE-specific terms (e.g., Top-Down Motor Milestone Test, Bidabe, MOVE Programme) and was limited to publications published after 1985, marking the onset of the MOVE programme’s development. Search strings were adapted to the indexing syntax of each database (Supplementary Material S1).

After removing duplicates, the remaining records were uploaded onto web-based systematic review management platform rayyan.ai. Duplicates were removed automatically and manually, and content in languages not spoken by the authors was translated using AI tools (DeepL v4.5 and v4.7, ChatGPT v4.0, Microsoft Translator v4.0), validated utilising parallel translation or by native speakers. All following steps of screening and data extraction process were concurrently and independently conducted by two reviewers, with inconsistencies at each stage resolved through discussion and consensus. To improve consistency, a sample of 30 articles was screened. The refined process was then applied for screening of all search results. Next, full texts were consulted according to predetermined criteria. Exclusion criteria at this stage were recorded and reported. Data extraction categories were collaboratively developed based on the scope of the research questions. Extracted information was categorised using an Excel spreadsheet.

The following data were extracted: authors; year, language, type of publication; geographic affiliation of the first author; relationship to MOVE (brief mention, one of several topics, main topic, conceptual foundation of MOVE). Publications cited within early publications of the MOVE programme were considered conceptual precursors of MOVE and only included in the content analysis.

Critical appraisal of individual sources of evidence was not performed, as the aim of this scoping review was to map the extent and nature of the existing literature, irrespective of methodological quality, in accordance with current scoping review guidelines [16]. Extracted data were analysed using structured qualitative content analysis based on predefined categories and synthesised through thematic mapping. Charts and graphs used to visualise synthesis were created using Microsoft Excel 2021. Interpretation and presentation were jointly agreed upon by the research team.

During the review process, the protocol was slightly adapted to better align with the aim of the scoping review. In the reference search, additional publications relevant for the conceptional foundation of the MOVE programme were included, even if published before 1986 [17,18,19,20,21,22,23,24,25,26,27]. These publications are only reported under results. In contrast, due to the large volume of the included literature, primary studies evaluating the MOVE programme and the Top-Down Motor Milestones Test (TDMMT) will be reported separately [28,29,30,31,32,33,34,35,36,37,38,39,40,41,42,43,44,45,46,47,48,49,50,51,52,53,54,55,56,57,58,59,60,61,62,63,64,65,66,67,68,69,70,71,72,73,74,75,76,77,78,79,80,81,82,83,84,85,86,87,88,89,90,91,92,93,94,95,96,97,98,99,100,101,102,103,104,105,106,107,108,109,110,111,112,113,114,115,116,117]. These publications are still considered in the descriptive synthesis of the results, and data relevant for other content categories are reported under results.

## 3. Results

**Figure 1:**
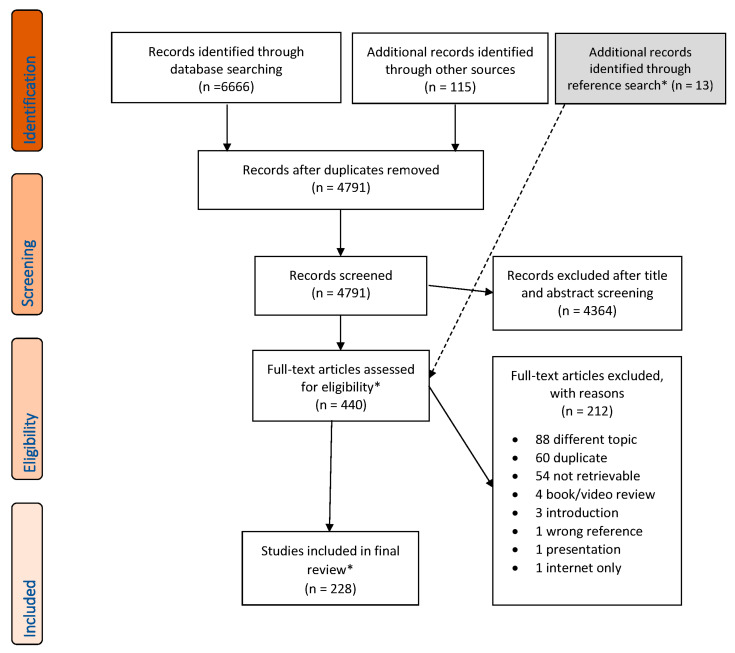
Prisma-ScR flow diagram of the selection process. * Thirteen publications on conceptual precursors of the MOVE programme were identified via reference search. Eleven retrievable articles were included in the content section, but not in the descriptive section of the result summary. Adapted from [15]. Licence: CC BY 4.0.

The literature search yielded 6666 articles; manual searching and private databases provided 115 additional publications. After the removal of duplicates, 4791 records were screened according to the inclusion and exclusion criteria, and 4364 were excluded. Thirteen publications relevant to the conceptual foundation of MOVE were added during the reference search. Out of the 440 resulting full texts, 212 were excluded for reasons outlined in the PRISMA-ScR flow chart (Figure 1, see Supplementary Table S2 for list of excluded literature and reasons for exclusion). A total of 228 publications were finally included in the scoping review.

**Figure 2:**
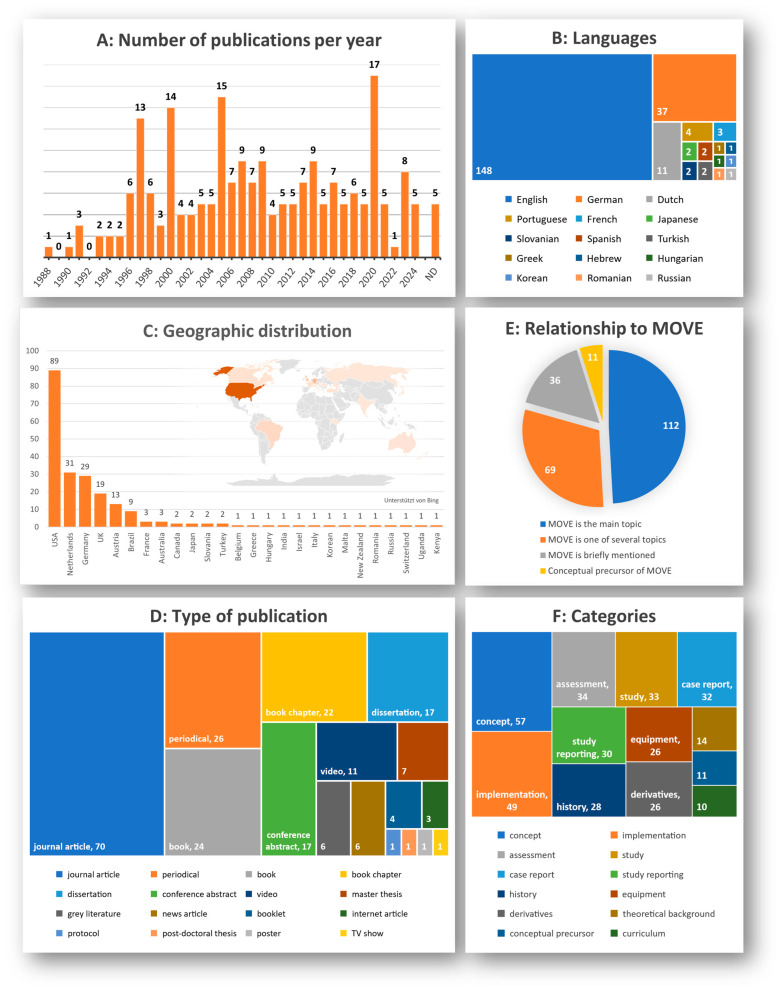
Publication characteristics. (**A**) The number of publications per years; (**B**) languages; (**C**) geographic distribution; (**D**) type of publication; (**E**) relationship to MOVE; (**F**) categories.

The included publications span from 1988 to 2024, with peaks linked to conferences or themed issues. They were written in fifteen different languages, predominantly English (148), German (37), and Dutch (11). The 132 unique first authors represent institutions across 26 countries, primarily in the United States, the Netherlands, the UK, Germany, Austria (see Figure 2C). Publications were categorised into 17 types, including 70 journal articles, 26 periodical articles, 24 books and 22 book chapters, and 26 theses in total (seven master’s, 18 doctoral, one post-doctoral). A total of 112 publications discussed MOVE as the main focus, 69 addressed it alongside other topics. Thirty-six publications mentioning MOVE only briefly were excluded from detailed content analysis [77,97,118,119,120,121,122,123,124,125,126,127,128,129,130,131,132,133,134,135,136,137,138,139,140,141,142,143,144,145,146,147,148,149,150,151]. Sources were further classified by their content related to the MOVE programme, with multiple categories possible per publication. Eleven sources cited in two early publications by Bidabe et al. [34,37] were classified as conceptual precursors [17,18,19,20,21,22,23,24,25,26,27]. Publication characteristics are illustrated in Figure 2, with further details presented in Supplementary Table S3.

### 3.1. History and Development

Twenty-eight publications documented the history and development of the MOVE programme [32,33,34,35,36,37,38,46,50,57,62,64,65,78,79,80,99,103,114,152,153,154,155,156,157,158,159,160]. From 1974, Linda Bidabe worked with students with severe disabilities in a day programme in Bakersfield, California, which later formed the Blair Learning Center [38]. At that time, U.S. legislation and court rulings required schools to provide appropriate education for all students [46,161].

Bidabe observed that her students were often non-ambulatory, required maximal assistance, and presented with various secondary complications [32]. Developmentally oriented approaches common at that time did not prevent further decline [32,33,34,37,38,46,50,65,153,155,158]. She recognised that students with better mobility also performed better in other areas like toileting, feeding, and alternative and augmentative communication, concluded that movement is the foundation for learning and personal dignity [34,78,79,162], and began developing a mobility-based intervention [38].

Bidabe initially interviewed parents to identify essential activities for an independent adult life of her students and analysed which locomotor skills were necessary to perform these activities [32,38,103]. Skills training was integrated into the school day to increase training opportunities and eliminate the need to transfer acquired skills from therapeutic to everyday contexts [34,99]. Together with colleagues, she developed mobility equipment and carried out a first successful pilot study in 1986, followed by a replication in Australia [37,38]. The early concept, *Standing Room Only*, focused on learning sequences using mobility equipment [38]. The programme was further refined and officially published under the name Mobility Opportunities Via Education (abbreviated M.O.V.E. or MOVE) in 1990 [34].

The manufacturing company Rifton Equipment (www.rifton.com) supported further development and dissemination of the equipment and the concept [38]. Following training activities, MOVE expanded across the United States and internationally, supported by non-profit organisations such as MOVE International and MOVE Europe [38,156,158]. By 2002, the MOVE curriculum was translated into 12 languages and implemented in 27 countries [35,36,154].

### 3.2. Concept

Fifty-seven articles described the concept of the MOVE programme [13,32,33,34,35,36,37,41,46,50,52,64,66,78,79,80,85,86,94,95,96,99,113,152,153,154,155,156,159,162,163,164,165,166,167,168,169,170,171,172,173,174,175,176,177,178,179,180,181,182,183,184,185,186,187,188,189]. MOVE is officially labelled as a ‘top-down, activity based curriculum designed to teach students basic, functional motor skills needed for adult life in home and community environments’ [34] (p. 2). Authors also characterise it as a task-oriented [99], naturalistic [155], ecological [153] approach, as a functional educational programme [32], or as ‘educational physiotherapy’ [173].

#### 3.2.1. Target Group

The original target group were non-ambulatory students over seven years, unless medical reasons hindered participation [34,78,79]. Later, the programme was expanded to infants and younger children, and students with orthopaedic needs [32,34].

#### 3.2.2. Structure

MOVE is individually planned and carried out in six steps [33,34,99,152,155,166,176,177,181]. (1) Testing: The child’s functional mobility skills are assessed through an interview with the participant and their caregivers, using the TDMMT. (2) Setting goals: Meaningful, age-appropriate activities are identified, based on the participant’s and their family’s needs and preferences, as well as activities that are physically demanding for the caregivers [165]. These goals foster independence, both in the near future and later in adulthood, and support a family-centred approach [41,85,94,113,162,169,170]. (3) Task analysis: Target activities are examined for their motor requirements, and relevant skills within the TDMMT are identified. (4) Measuring prompts: The type and amount of support currently required by the child to perform the target activities are defined. (5) Reducing prompts: Gradual reduction in the support is planned, in line with the student’s expected learning progress, and documented and monitored in prompt reduction plans for sitting, standing and walking, and arm use for sitting and standing or walking [34,46,50,78,79,95,96,156,185]. For participants with degenerative disorders, prompts may instead need to be increased to maintain active participation in daily activities [34,156]. (6) Teaching skills follows four stages: in the *acquisition stage*, the selected skills are addressed in one-on-one situations in specific MOVE lessons; in the *fluency stage*, they are practised in relevant everyday routines in the natural environment [152,158,165,166,177,181], until the participant progresses to the *maintenance stage*, where skill performance is consolidated. Acquired skills are transferred to other situations and settings, such as the participant’s home, during the *generalisation stage*. This approach promotes participation in daily life [180] and supports the generalisation of new skills [34,78,79].

#### 3.2.3. Teaching Methods

To enable motor learning, participants practise on the just manageable level [34], receiving only necessary support [34,50,78,79]. Teaching methods for every skill are described in the training materials, including two specific techniques. In *shaping*, brief support is given to help assume a position, which is then gradually reduced. In *guiding*, participants are supported through a movement in a way that allows for active participation—for example, rising to stand with their weight positioned over their feet—so that the support can gradually be withdrawn [34,35,36,78,79].

#### 3.2.4. Teamwork

Bidabe’s expression ‘It takes a team to MOVE’ [34,159] indicates that teamwork is a central characteristic of the programme. The participant and their primary caregivers are core members of this team, while therapeutic and educational staff, including support staff, form the inner MOVE team responsible for daily implementation. Additional members, such as medical doctors, may be involved as needed [34,78,79]. The team is characterised as collaborative [32,156,163], inter- [41,179], or transdisciplinary [113,155,176]. This approach supports working towards common goals and integrating practice into daily activities. The roles of the family, school assistants, and physiotherapists within the team were discussed in three publications [85,190,191].

In one publication on postural care, the MOVE programme was criticised for inadequately addressing this issue while emphasising short-term goals [171].

### 3.3. Assessment

Thirty-four publications discussed the TDMMT [32,33,34,35,36,69,75,76,78,79,98,102,104,152,155,158,170,172,174,183,184,188,192,193,194,195,196,197,198,199,200,201,202,203]. Being a criterion-referenced measure, the TDMMT assesses functional mobility skills across 16 categories such as sitting, movement while sitting, standing, and walking forward. It contains 74 items ordered into four *success levels*, (Grad Level and Levels I–III), ranging from tolerating basic positions and passive movements at Level III to independent locomotion at home and minimal outdoor assistance at Grad Level [32,34,195,199].

The TDMMT was developed to customise the approach and track participants’ progress [183,195,201] and not as an outcome measure [195]. It has been characterised as an assessment of movement skill capacity and performance, within the ICF-Domains of Activity and Participation [200]. According to Bidabe [34], the assessment is used in Step one (assessment of current skills) and Step three (identification of locomotor skills needed for personal goals). It is conducted retrospectively based on caregiver or team input, to identify the highest skill consistently observed in daily life (performance). If this information is not available, the skill can be assessed directly in the test situation (capacity). The TDMMT uses hierarchical scoring, so that all items below the identified item are considered achieved.

### 3.4. Conceptual Precursors

**Figure 3:**
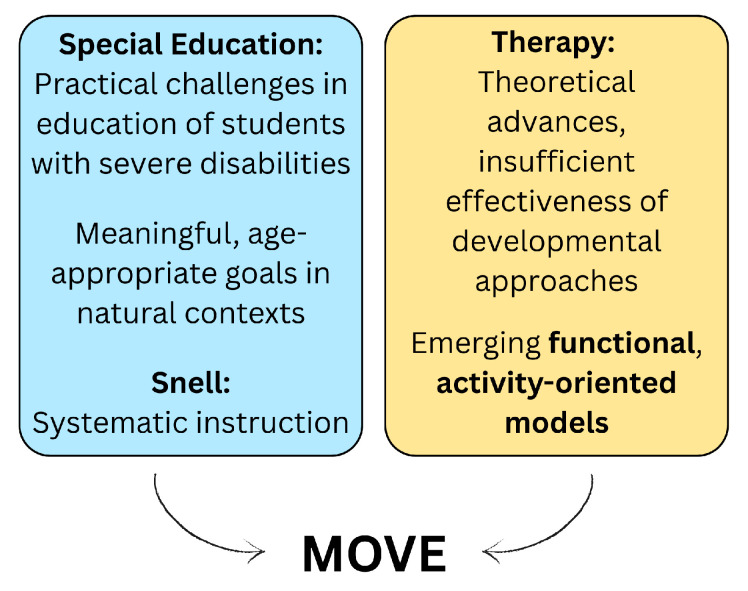
Conceptual precursors of the MOVE programme.

MOVE was influenced by several conceptual precursors from education and physiotherapy, as documented in two early publications by Bidabe [34,37] (see Figure 3). Of these thirteen references, eleven could be retrieved [17,18,19,20,21,22,23,24,25,26,27]. Two articles emphasised the need for chronological-age-appropriate, functional goals implemented through the principle of partial participation [17,21]. Contextual programming involves teaching skills within meaningful, everyday contexts, in contrast to standardised therapeutic settings, to promote skill generalisation and faster skill acquisition [24].

In order to provide appropriate education for students with severe disabilities, Snell et al. proposed a concept of systematic instruction [27]. They argued that ‘all people with severe handicaps can learn, although learning is likely to be slow’ [27], and outlined a procedure for assessment and curriculum development in five iterative phases [21]: (1) multidisciplinary assessment, using norm-referenced or criterion-referenced tests, and ecological inventory of current and future environments; (2) identification of short- and long-term goals; (3) analysis of behaviour and tasks; (4) specification of intervention techniques and data collection designs; and (5) collection of baseline and intervention data. Snell et al. referred to this approach as being Top-Down in contrast to developmental Bottom-Up models [27]. Implementation of this procedure with regard to motor abilities recommended instructional strategies such as positioning techniques to normalise muscle tone and the facilitation of coordinated movement patterns within natural contexts, aiming to support functional outcomes in everyday activities [22,23].

The report of adverse effects of reclined posture in sitting, like the promotion of the extensor thrust and a non-functional seating position [25], informed the front-leaning position of the MOVE equipment [34].

### 3.5. Theoretical Background

The MOVE programme was developed through practical experience; the original authors did not provide an underlying theory [95,96,99]. Fourteen publications [29,30,46,92,95,96,99,115,155,162,204,205,206,207] retrospectively proposed a basis in:

1. Dynamic systems theory: Motor control emerges from the interaction of individual, task, and environment [29,204,205,208];
2. Task-oriented approaches: Focus on functional use of movement, task-analysis, and natural environments [99];
3. Motor learning concepts: Self-initiated movements combined with prompt fading and environmental adaptations support skill acquisition [30,95,96,99,204,205,206,207];
4. Vygotsky’s Zone of Proximal Development: Scaffolding principles applied to mobility training [30,204,205].

**Figure 4:**
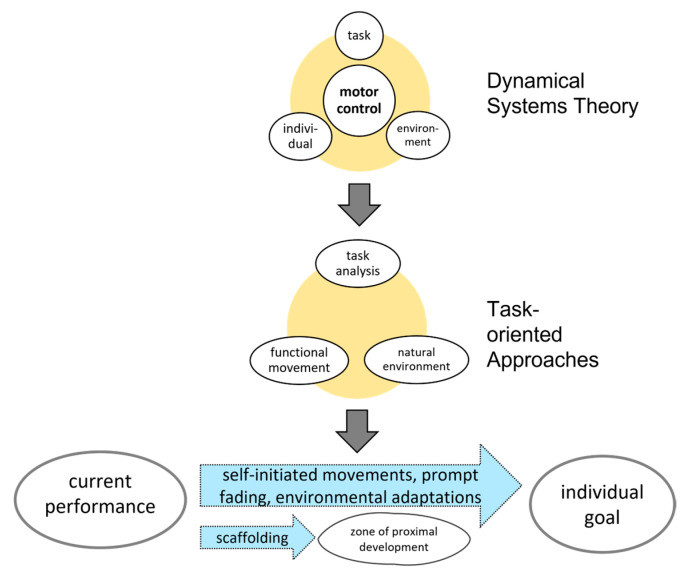
Retrospectively developed theoretical background of the MOVE programme.

From Thompson’s perspective, these features classify MOVE as a task-oriented approach, consistent with other educational and rehabilitation models [209,210,211] (see Figure 4).

### 3.6. Implementation

Forty-nine publications referred to programme implementation [32,34,35,36,47,49,52,53,56,57,58,63,65,72,78,79,80,81,82,85,108,152,156,159,162,170,173,181,182,189,190,191,212,213,214,215,216,217,218,219,220,221,222,223,224,225,226,227,228].

Official publications and training materials [34,35,36,78,79], supplemented by standardised worksheets [217,229], provide extensive guidance on the structured approach, and include strategies for sustainable outcomes for participants and safe handling techniques [78,79,218]. Additional audio–visual resources have been developed for specific topics such as incorporating MOVE within Individualised Educational Plans [57,58,212,218,228].

These resources are regularly used in structured in-person MOVE trainings for both professionals and laypersons. In the US and UK, two-day courses qualify participants as MOVE Practitioners and Senior Practitioners, respectively. In German-speaking countries, both courses are combined in one single four-day practitioner course. Experienced practitioners may further qualify for roles as MOVE trainers, while some take on facilitation roles as MOVE counsellors [79].

Quality insurance is enhanced through Bronze, Silver, Gold, and Centre of Excellence marks, which encompass an evaluation of curriculum, documentation, equipment, and training. The Centres of Excellence additionally serve as model sites where interested parties can observe MOVE practices in action [220].

Implementation has been reported from educational and therapeutic settings, residential care facilities, and hospitals around the world [32,47,49,53,57,63,65,72,81,82,83,156,159,170,189,213,214,215,216,219,221,222,223,224,225,226,227]. Although the programme recommends focusing on selected items of the TDMMT, additional teaching material gives the impression of general mobility activation throughout the day [212]. In one case, MOVE was implemented using various stations for balance, strength, and coordination exercise [215], and in another case as a half-day programme separate from the classroom, with goals across all categories of the TDMMT [52].

### 3.7. Equipment

Twenty-six publications discussed the use of equipment [32,33,34,35,36,37,38,57,59,60,61,62,69,70,78,79,80,99,158,163,164,193,198,230,231,232]. Prototypes by Bidabe and colleagues were: a Front-Leaning Chair with adjustable support for head, trunk, legs; a Mobile Stander—a standing frame equipped with large wheels allowing for self-propulsion; and a Front-Leaning Walker with adjustable prompts for upright standing and supported stepping [33,34,37]. Rifton Equipment subsequently produced and refined these devices and added a universal seating frame [32,38,80].

Equipment is seen as an instructive aid: instead of replacing missing skills, it supports active motor learning while reducing caregiver burden [34,35,36,37,78,79,80]. The front-leaning feature of the devices supports active movement. Support is adjustable to enable prompt reduction as the participant progresses [34,78,79,99,163] so that some devices may become unnecessary over time [34,38]. Other manufacturers’ equipment and standard furniture can equally be used if they allow for prompt adaptation [62].

Mobility equipment is used to support independence, exploration, spatial understanding, and choice-making of participants [164]. The increased use of gait trainers, also known as supportive stepping devices, in school settings has been partly attributed to the development of the MOVE programme [230].

### 3.8. Adoption in Educational Curricula

Ten publications addressed the role of the MOVE programme for educational curricula [152,172,173,175,213,222,233,234,235,236]. MOVE was presented as one relevant intervention to achieve the appropriate education of students with cerebral palsy and CP-like conditions: promoting the achievement of the highest possible level of mobility may help them to gain control over their environment [175]. In this context, interventions should focus on goals relevant to students, caregivers, and teachers, and follow a top-down approach with focus on social participation [152]. The MOVE programme has also been seen to be useful in assessing students with visual or hearing impairment, or deaf-blindness in regard to general physical education [172].

In Russia, Romania, and Australia, MOVE has been recommended to supplement general national curricula [173,233,234,236], and in Denmark and the UK to promote school development and reorganisation [213,222].

### 3.9. Adaptations and Derivatives

Twenty-six publications reported adaptations of the original MOVE programme and the TDMMT for other target populations, settings, and activity situations [59,60,61,62,74,75,76,102,104,158,193,198,229,231,237,238,239,240,241,242,243,244,245,246,247,248]. MOVE for Adults addresses the needs for adults with disabilities with minor changes to the six steps of execution. Here, the TDMMT includes additional items within the categories walking on uneven ground, up slopes, and down slopes [62].

The MOVE Hygiene and Toileting program follows six steps to promote independence in toileting for adults with disabilities, including transfers and adjusting clothing [193]: (1) skill assessment via interview and the Top-Down Toileting Assessment, in which TDMMT’s advanced walking categories are replaced with Hand and Arm Use and Communication for Toileting; (2) selection of the team and toileting routine; (3) preparation of the toileting environment and completion of the assessment; (4) task-analysis; (5) teaching strategy; and (6) teaching and generalising the skills [193,245]. The MOVE Toileting Care Program serves similar purposes for adults with disabilities and elderly people [198]. A Toileting Chair and a Changing Station were created by Rifton Equipment [59,60,61,193,231]. These separate programmes have now been integrated into regular MOVE Practitioner training in the United States (Christine Sarnacki, MOVE International, personal communication, 2025).

The MOVE programme has been considered for non-ambulatory students with sensory and multi-sensory disabilities to support the development of mobility and orientation skills [129]. MOVE has further been proposed for use with elderly populations [158,241]; however, practical implementation is currently not reported.

The TDMMT has undergone several adaptations. For use as an outcome measure, Putten et al. adjusted the item order and allocatio, and omitted two items identified as redundant [102,104]. Within the Mobilitätstest für alte Menschen (Mobility Test for the Elderly, MOTA), categories, items, and item order were modified for use with elderly individuals [237]. The MOTA subsequently served as a template for the Mobility Test for Patients in Acute Care (MOPTA) [239]. Additionally, the Functional Assessment of Students with Severe Disabilities modelled two mobility domains after the TDMMT [194].

See Table 1 for an overview of the MOVE programme.

## 4. Discussion

This scoping review aimed to comprehensively map and describe the literature on the MOVE programme and its relevance for non-ambulatory individuals with cerebral palsy. A broad range of publications have been published between 1988 and 2024, originating mainly from the United States and Europe, and encompassing both peer-reviewed and non-peer-reviewed sources.

Extensive information was gathered regarding MOVE development, concept, conceptual precursors, theoretical foundation, and role of assessment and equipment. MOVE was developed during a period of conceptual change in special education and therapy. The lack of established, age-appropriate concepts created considerable challenges in educating students with severe disabilities, who gained access to education [219]. Although Snell offered a coherent and structured framework for implementing these ideas in education [27], translation into practice remained limited [22,23]. Against this background, Bidabe developed the MOVE programme, drawing on Snell’s framework, to create a functional motor approach for school settings. MOVE thus marked a shift from traditional interventions towards participation-focused education [99].

The MOVE concept incorporated features such as family-centred goals and meaningful activities practised in natural settings, as well as the emphasis on teamwork, which are reflected in current clinical guidelines [9]. By integrating adapted equipment, such as mobile standers, walkers, and supportive seating, MOVE likely contributed to the broader adoption of such devices, particularly in special schools [230].

The TDMMT used in MOVE was primarily designed to guide and track individualised programming, reflecting parent input, rather than to serve as a validated outcome measure [38,195]. For this reason, the TDMMT should not be used as an outcome measure in the evaluation of motor programmes, including MOVE; evaluations should instead employ assessments with established psychometric validity.

At the time of MOVE’s development, theoretical models underpinning functional approaches were still emerging, and Bidabe developed MOVE from a practical rather than academic background. This resulted in a lack of explicit theoretical foundation. Retrospective analysis aligns MOVE with the dynamic systems approach to motor control and motor learning principles. MOVE has therefore been classified as a functional, activity-based, task-oriented intervention [99].

Implementation is mainly guided by official publications, worksheets, and audio–visual material, with a six-step process guiding individual programming [34]. However, some reports indicate deviations, such as implementation only in isolated practice sessions or using it for generic activation without individualised goals. These deviations represent a shift from the programme’s core philosophy of contextual learning and significantly reduce practice intensity and motivation in the participants. Given that individuals with complex disabilities often struggle to generalise motor skills across different settings, isolated training may result in lower functional gains and a failure to transfer skills to daily routines. This not only leads to less meaningful outcomes but may also cause a perceived lack of progress, eventually resulting in the discontinuation of practice. This suggests the need for clearer communication of its core characteristics to ensure intervention fidelity [52,215].

MOVE has been applied in diverse settings and among individuals with disabilities across the lifespan [47,115,193]. Systematic implementation for older populations has been proposed [241], but not yet been reported.

Integration of MOVE into educational curricula has been discussed by researchers, practitioners, and educational authorities. Tensions exist between national, standardised curricula and the individual needs of students with disabilities. Within this context, MOVE is recognised as a relevant framework for non-ambulatory students [175,233,234,236].

MOVE has been criticised for paying insufficient attention to long-term goals and postural alignment, reflecting a broader tension between approaches focusing on postural quality and those prioritising functional mobility [171]. Ensuring safe practise and preventing adverse effects remains essential. Within MOVE, physiotherapists monitor orthopaedic risks and supervise practise, so alignment is managed through clinical oversight rather than explicit programme components [78,79]. Moreover, as in individuals without disability [249], postural quality may improve during the acquisition of new skills [250,251], reflecting Bidabe’s motto: ‘move first, pretty later’. Functional, goal-directed activity may therefore support postural control by helping children develop more stable and adaptive movement strategies.

It is a fundamental premise, supported by this review, that active participation and functional motor learning are not only essential but also achievable for individuals—across childhood, adolescence, and adulthood—with non-ambulatory cerebral palsy and similar complex disabilities. Recognising this possibility is a crucial step in overcoming common therapeutic barriers. For the MOVE programme to be successfully implemented, its core ingredients must be consistently integrated: its primary strength lies in the synthesis of individually meaningful participation goals with continuous, long-term practice in everyday environments. The use of assistive mobility devices, especially in non-weight-bearing individuals, facilitates physical activity and enables motor learning while simultaneously reducing caregiver burden.

In line with clinical guidelines [9], practice should prioritise functionality over movement quality, as active engagement is the overriding goal and quality often improves through consistent practice [251]. While intervention in childhood helps maximise functional mobility, introducing or continuing the programme in adulthood is paramount to prevent functional decline, mitigate reduced participation, and avoid long-term health complications. To date, the MOVE programme has primarily been utilised in educational and paediatric care settings. Although successful application in hospital environments and adult care has been reported, it remains notably underutilised in these contexts. A significant gap exists in vocational and community settings for adults with disabilities. Given the high levels of sedentary behaviour in these environments [13], expanding MOVE into adult care represents a vital opportunity to foster functional activity during daily routines. Furthermore, the adaptive application of MOVE principles could be pursued for elderly individuals with restricted mobility to mitigate age-related functional decline.

Since MOVE was introduced and much of the reviewed literature was published, research, concepts, and motor interventions in the field of childhood-onset disabilities have advanced considerably. Further research on evidence-based practice and implementation of the MOVE programme should therefore focus on several key aspects: reviewing and, where necessary, expanding the existing evidence base; investigating the mechanisms in which MOVE produces meaningful outcomes; examining patterns in how different participants respond to the programme; and identifying facilitators and barriers to implementation. It will also be valuable to explore in detail how MOVE aligns with current theoretical knowledge and practice guidelines and whether conceptual updates are warranted.

## 5. Limitations

This article represents the first attempt to characterise the MOVE programme through a comprehensive map of the existing literature. The combined search strategy, using both specific and relatively broad search terms, successfully identified a wide range of the literature. The use of English-language search terms may have, however, introduced a language-related publication bias. Although MOVE is reported to be used in multiple countries, it was only partly possible to retrieve the literature reflecting this circumstance. Most articles stem from the United States and European countries, likely associated with the activities of MOVE International and MOVE Europe, as well as the work of researchers and practitioners based there. The low number of articles documenting the use of the MOVE programme in LMICs might be explained by the need for specialised equipment, limited dissemination, reduced publication activity in these countries, or search bias.

Peer-reviewed articles constitute a relatively small proportion of the reviewed literature. This is partly due to the broad scope of this review, which included publications from periodicals, theses, and other sources, and may also indicate that the MOVE programme has received greater attention in clinical practice than in scientific research. The results and discussion presented here are primarily based on peer-reviewed and scientific literature, as well as official publications.

A further limitation of the search strategy is the inclusion of sources from own collections, which may reduce the reproducibility and transparency of the review. While such sources were deemed relevant and important, their accessibility to external researchers can be limited.

## 6. Conclusions

In conclusion, the MOVE programme provides a structured yet flexible interdisciplinary framework that enables functional mobility and active participation for individuals with complex, non-ambulatory disabilities. Crucially, this review reinforces the premise that functional motor learning is achievable across the entire lifespan when practice is integrated into natural, goal-oriented environments. While MOVE is partially established in educational settings, there is an urgent need to expand its implementation into adult services—particularly residential homes and vocational settings—to combat the high levels of sedentary behaviour in these populations.

To ensure meaningful outcomes, future implementation must maintain high fidelity to the programme’s core ingredients, avoiding isolated practice sessions in favour of contextual learning. Finally, as the current body of literature consists of diverse sources and a retrospectively developed framework, further rigorous empirical investigations are warranted to validate the programme’s underlying mechanisms and its long-term efficacy across various stages of life.

## Figures and Tables

**Table 1:** Main findings.

History and development	lack of educational programmes for students with severe disabilities	Bidabe’s practical experience with students with severe disabilities	pilot programme with positive outcomes	publication of the MOVE programme
Concept	target group: children and adolescents with non-ambulatory disabilities; expanded to younger children and	implementation in six steps: testing, setting goals, task-analysis, prompt assessment, prompt reduction, teaching skills	family-centred team-approach; individual, client-chosen goals; practice embedded into natural settings; motor learning approach; use of assistive mobility devices as learning tools	criticised by some authors for pivoting away from postural care
Assessment	TDMMT developed in cooperation with parents	16 functional mobility categories	72 items across 4 levels of success	programming tool, not intended as outcome measure
Conceptual precursors	education: age-appropriate education of students with severe disabilities	education: partial participation, contextual programming, scaffolding	education: systematic instruction (M. Snell)	physiotherapy: functional, activity-oriented approaches
Theoretical background	retrospectively developed	dynamic systems theory	task-oriented interventions	motor learning concepts
Implementation	guided by training material and courses	quality insurance through quality marks	implemented across various settings	deviations from concept reported
Equipment	based on prototypes developed by Bidabe’s team	front-leaning chair, mobile stander, front-leaning walker	developed as instructive aids with easy prompt adaptation	supports independence, exploration, and choice-making
Adoption in educational curricula	adoption in various countries to supplement national curricula	integrated into regional and local curricula	considered for students with sensory impairments	adopted in physical education curriculum
Adaptations and derivatives	Standing room only: pilot study focussing on equipment	MOVE for adults—Mobility Opportunities via Experience	MOVE Hygiene and Toileting programme: independence in hygiene	MOVE for the elderly: proposed but not implemented

## Data Availability

No new data were created or analysed in this study. Data sharing is not applicable to this article.

## Supplementary Materials

The following supporting information can be downloaded at https://www.mdpi.com/article/10.3390/children13020292/s1, Table S1: Databases; Table S2: Excluded literature and reasons for exclusion; Table S3: Included literature; Material S1: Search string PubMed via EbscoHost.

## Abbreviations

The following abbreviations are used in this manuscript:

GMFCS	Gross Motor Function Classification System—expanded and revised
HIC	High-income countries
LMIC	Low- and middle-income countries
MOTA	Mobilitätstest für alte Menschen [Mobility Test for Elderly]
MOPTA	Mobility Test for Patients in Acute Care
MOVE, M.O.V.E.	Mobility Opportunities Via Education
PRISMA-ScR	Preferred Reporting Items for Systematic reviews and Meta Analysis extension for Scoping Reviews
TDMMT	Top-Down Motor Milestones Test
