# Supplementary material for: Promoting Functional Mobility in Individuals with Non-Ambulatory Cerebral Palsy: A Scoping Review of the MOVE Programme
Source: Children (Basel). 2026 Feb 20;13(2):292. doi: 10.3390/children13020292 (PMC12939002; doi:10.3390/children13020292)
Supplement: Supplementary file 1 [file children-13-00292-s001.zip › Schomerus supp mat S1 search string PubMed via EBSCOHost.pdf]

((("locomotor rehabilitation" OR "locomotor training" OR "locomotor therapy" OR "ambulation training" OR "gait training” OR "gait trainer" OR "assisted stepping") AND ("non-ambulant" OR "non-ambulatory" OR "mobility impairment" OR ((profound OR severe) AND (multiple OR physical) AND (disabilit* OR impairment)))) **OR** ((Linda AND Bidabe) OR (Bidabe AND LOLLAR) OR (Bidabe AND Move) OR (Barnes AND Move) OR (Whinnery AND Move) OR (Putten AND Move) OR (Mobility AND Opportunities AND Via AND Education) OR (Top AND Down AND Motor AND Milestone AND Test) OR (TDMMT))) **AND** (yearpublished > 1985)

Supplementary material S1: Search string for PubMed MEDLINE via EBSCOhost
