# Supplementary material for: Promoting Functional Mobility in Individuals with Non-Ambulatory Cerebral Palsy: A Scoping Review of the MOVE Programme
Source: Children (Basel). 2026 Feb 20;13(2):292. doi: 10.3390/children13020292 (PMC12939002; doi:10.3390/children13020292)
Supplement: Supplementary file 1 [file children-13-00292-s001.zip › Schomerus supp table S1 databases.pdf]

Database	Date	Results	Notes
Cinahl via EBSCOhost	08 May 2024	276	
ERIC via EBSCOhost	08 May 2024	32	
PSYNDEX literature with PSYNDEX tests via EBSCOhost	09 May 2024	4	
Education Source via EBSCOhost	09 May2024	552	Limited to 250 results per search term
Pubmed MEDLINE via EBSCOhost	09 May 2024	526	Limited to 250 results per search term
APA PsychInfo via EBSCOhost	09 May 2024	103	
APA Psycharticles via EBSCOhost	09 May 2024	294	Limited to 250 results per search term
Academic Search Ultimate via EBSCOhost	09 May 2024	1623	Limited to 250 results per search term
SCOPUS	09 May 2024	284	
BASE	10 May 2024	439	Limited to 250 results per search term
ProQuest	10 May 2024	932	Limited to 250 results per search term; excluding the terms “rugby” and “Ugaritic”
LIVIVO (bibnet.org, current contents, DissOnline, ETHMED, EZB, NLM, ZB MED, SOMED, Verlagsdaten, ZB Sport)	10 May 2024	0	
Google Scholar	12 May 2024	1215	Only specific search terms; limited to 250 results per search term
WorldCat	16 May 2024	386	Limited to 250 results per search term

Supplementary Table S1: Included databases and search engines
