# Supplementary material for: Promoting Functional Mobility in Individuals with Non-Ambulatory Cerebral Palsy: A Scoping Review of the MOVE Programme
Source: Children (Basel). 2026 Feb 20;13(2):292. doi: 10.3390/children13020292 (PMC12939002; doi:10.3390/children13020292)
Supplement: Supplementary file 1 [file children-13-00292-s001.zip › Schomerus supp table S2 excluded literature.pdf]

No	Reason for exclusion	Author	Year	Title	Journal	Volume	Pages	Publisher	Location
1	book review		1999	Movement Skill Assessment	Palaestra	15	53		
2	book review		2004	Review: MOVE (Mobility Opportunities Via Education) Videotapes	Zero to Three	24	53–54		
3	book review			No Ordinary Move (Book review)	Palaestra	18	56		
4	book review	Low, Sheryl A.	2007	CHILDREN WITH SEVERE DISABILITIES AND THE MOVE CURRICULUM--FOUNDATIONS OF A TASK ORIENTED THERAPY APPROACH (Book Review)	Physical & Occupational Therapy In Pediatrics	27	97--102		
5	different topic	Afzal, Farjad; Ahmed, Hafiz Ijaz; Asim, Hafiz Muhammad; Rasul, Akhtar; Islam, Asif	2015	Effects Of Universal Exercise Unit Combined With Conventional Combination Therapy On Gross Motor And Functional Skills In Spastic And Athetoid Cerebral Palsy Children	Int j med appl health	3	28–34		
6	different topic	Bailes, Amy F.; Greve, Kelly; Schmitt, Laura C.	2010	Changes in two children with cerebral palsy after intensive suit therapy: a case report	Pediatric physical therapy	22	76--85		
7	different topic	Barnes, Ellie; Hillier-Moses, Gemma; Murray, Helen; Stevinson, Clare; Franks, Hester A.; Gossage, Lucy	2023	Evaluation of the MOVE online exercise programme for young people aged 13-30	Supportive care in cancer	31	377	Springer International	Nottingham
8	different topic	Bosca, Concha Aldomar; Lopez Sanchez, Esperanza; Ros Pallares, Immaculada; Aldomar, Arancha Tejero; Estrela, Eduardo Signes; Blanco, Javier Segovia		La accesibilidad al medio fisico	Informacio Psicologica				
9	different topic	Brix, Olga	2015	CPCHILD(r)-DE: Ein Instrument zur Messung der Fähigkeiten, des Wohlbefindens und der gesundheitsbezogenen Lebensqualität von Kindern und Jugendlichen mit Behinderung					München
10	different topic	Brug, Annet ten; van der Putten, Annette A. J.; Vlaskamp, Carla	2013	Learn and apply: using multi-sensory storytelling to gather knowledge about preferences and abilities of children with profound intellectual and multiple disabilities--three case studies	Journal of intellectual disabilities	17	339--360		
11	different topic	Catama, Bryan V.; Domalanta, Ann B.	2024	Empowering and Equipping Parents with the Basic Intervention Skills in Handling their Children with Special Learning Needs	Texila International Journal of Academic Research	11	68--76		
12	different topic	Cay, Evgin; Sivrikaya, Tugba; Cetin, Muzeyyen Eldeniz		Rehberlik Araştırma Merkezlerinde Eğitsel Değerlendirme Yapan Personelin Çoklu Yetersizliği Olan Bireyleri Değerlendirme Süreçlerine İlişkin Görüşleri	Van Yüzüncü Yıl Üniversitesi Eğitim Fakültesi Dergisi	19	1047–1067	Van Yuzuncu University	
13	different topic	Chard, Melissa	2014	Polyhandicap et évaluation cognitive: apports du paradigme d'habituation visuelle					
14	different topic	Chen, Dingfang; Wu, Yuefeng; Li, HaiYing; Pan, Xue; Zhou, Jin	2022	Treatment on patients with spastic cerebral palsy in the past 30 years: A systematic review and bibliometric analysis	Medicine	101	e30535	Lippincott Williams & Wilkins	Changsha
15	different topic	Chow, Daniel H. K.; Chen, Wilson H. W.; Tam, Simone S. M.	2020	A Video-Based Classification System for Assessing Locomotor Skills in Children	Journals of sports science & medicine	19	585–595		
16	different topic	Cross, Alice Frazeur; Traub, Elizabeth K.; Hutter Pishgahi, Lois; Shelton, Gen	2004	Elements of Successful Inclusion for Children with Significant Disabilities	Topics in Early Childhood Special Education	24	169–183	Sage Publications	
17	different topic	Cytera, Chirine; Thyed, Ute	2021	"Ich finde, das ist halt in vielen Punkten im Leben wichtig" - Subjektive Vorstellungen junger Erwachsener von Teilhabe. ["I think it is Important in Many Aspects in Life" - Young Adults' Perception of Participation.]	Die Rehabilitation	60	384–392		
18	different topic	Donaghy, Tana Eileen	2015	Instruction of Students With Disabilities Cognitively Functioning Below Age 2					
19	different topic	Doty, Antonette	1999	ASSISTVE TECHNOLOGY IN OKLAHOMA PUBLIC SCHOOLS: A SERVICE DELIVERY MODEL FOR RURAL SCHOOLS	American Council on Rural Special Education.		81	Citeseer	
20	different topic	Effgen, Susan K.	2007	Updated competencies for physical therapists working in schools	Pediatric physical therapy	19	266–274		

21	different topic	Farrell, Michael	2004	Inclusion at the Crossroads: Special Education--Concepts and Values				David Fulton Publishers	Oxford
22	different topic	Fernes, Jose Marco Manuel; Miler, Monique de; van der Merwe, Elna	2022	The effect of a motor intervention programme for learners identified with moderate to severe intellectual disabilities	Heliyon	8	e11165		
23	different topic	Forster, S.; Iacono, T.	2014	The nature of affect attunement used by disability support workers interacting with adults with profound intellectual and multiple disabilities	Journal of Intellectual Disability Research	58	1105--1120		
24	different topic	George, Ciaran; Levin, Wendy; Ryan, Jennifer M.	2020	The use and perception of support walkers for children with disabilities: a United Kingdom survey	BMC Pediatrics	20	1–11	BioMed Central	London
25	different topic	Gerken, Lauren; Baumgartner, Lisa M.	2023	Do They Even Need ``Adult'' Education? A Programming Critique of Day Habilitation	New Horizons in Adult Education and Human Resource Development	35	51--55		
26	different topic	Hands, Beth P.	2002	How can we best measure fundamental movement skills?					
27	different topic	Harvey, Adrienne; Robin, Jonathan; Morris, Meg E.; Graham, H. Kerr; Baker, Richard	2008	A systematic review of measures of activity limitation for children with cerebral palsy	Developmental medicine and child neurology	50	190--198		
28	different topic	Hildeband, Mary; Neufeld, Peggy	2009	Recruiting older adults into a physical activity promotion program: Active Living Every Day offered in a naturally occurring retirement community	The Gerontologist	49	702--710		
29	different topic	Hildebrand; James Stuart	2014	Development of a Control System for a Power Wheelchair Trainer					Allendale
30	different topic	Holland, Peter	08/03/2000	Closing Remarks					
31	different topic	Hüche Larsen, Helle; Frisk, Rasmus Feld; Willerslev-Olsen, Maria; Nielsen, Jens Bo	2021	Motor-learning based activities may improve functional ability in adults with severe cerebral palsy: A controlled pilot study	NeuroRehabilitation	48	273–283	IOS Press	
32	different topic	Hull, Jennifer Ruhl	2005	General classroom and special education teachers' attitudes toward and perceptions of inclusion in relation to student outcomes					
33	different topic	Hutchinson, Christine	2013	Positive Health: A Collective			1		
34	different topic	Hwang, Jongseok	2021	Effect of an End-effector Type of Robotic Gait Training on Stand Capability, Locomotor Function, and Gait Speed in Individuals with Spastic Cerebral Palsy	Journal of The Korean Society of Physical Medicine	16	123--130		
35	different topic	Jackman, Michelle; Sakzewski, Leanne; Morgan, Catherine; Boyd, Roslyn N.; Brennan, Sue E.; Langdon, Katherine; Toovey, Rachel A. M.; Greaves, Sue; Thorley, Megan;	2022	Intervenções para promover função física de crianças e jovens com paralisia cerebral: diretriz internacional de prática clínica.	Developmental medicine and child neurology			Blackwell	Brisbane
36	different topic	Jackman, Michelle; Sakzewski, Leanne; Morgan, Catherine; Boyd, Roslyn N.; Brennan, Sue E.; Langdon, Katherine; Toovey, Rachel A. M.; Greaves, Susan; Thorley, Megan;	2022	Interventions to improve physical function for children and young people with cerebral palsy: international clinical practice guideline	Developmental medicine and child neurology	64	536–549	Blackwell	Brisbane
37	different topic	Jekosch, Sabine; Brach, Michael; Schulz, Henry; Dierbach, Oskar; Heck, Hermann	1998	Prüfung der Gütekriterien bei einem neuen Mobilitätstest für hochbetagte Menschen [Examination of the Psychometric Properties of a New Mobility Test for the Very Elderly]			103–109		
38	different topic	Karp, Gary	2008	Life on Wheels: The a to Z Guide to Living Fully with Mobility Issues				Springer Publishing	
39	different topic	Kelsey, Elizabeth	2024	'All kids belong'	Telegraph - Herald				Dubuque

40	different topic	Kenyon, Lisa K.; Farris, John; Brockway, Kaelee; Hannum, Nanette; Proctor, Kevin	2015	Promoting self-exploration and function through an individualized power mobility training program	Pediatric physical therapy	27	200--206		
41	different topic	Kwon, Jeongyi		Dieagnostic Evaluation and Rehabilitation in Children with Intellectual Disabilities					
42	different topic	La Cruz, S. Perez de	2007	[Duplikat] Tratamiento y evaluación de las deficiencias motoras en el aula. Guía práctica para padres	Revista Iberoamericana de Fisioterapia y Kinesiologia	10	55–64	Elsevier	
43	different topic	La Cruz, S. Perez de	2007	Tratamiento y evaluación de las deficiencias motoras en el aula. Guía práctica para padres	Revista Iberoamericana de Fisioterapia y Kinesiologia	10	55–64	Elsevier	
44	different topic	Lacey, Penny; Ouvry, Carol	2013	People with profound & multiple learning disabilities. A collaborative approach to meeting complex needs				Routledge	London
45	different topic	Lancioni, Giulio E.; Singh, Nirbhay N.; O'Reilly, Mark F.; Sigafoos, Jeff; Alberti, Gloria; Campodonico, Francesca	2016	Case Studies of Technology-aided Interventions to Promote Hand Reaching and Standing or Basic Ambulation in Persons with Multiple Disabilities	Perceptual & Motor Skills	122	200–219		
46	different topic	Lopez, Fernandez; Molina, Pelegrin		Necesidadaes Educativas Especiales del alumnado con discapacidad fisica					
47	different topic	Maes, Bea; Nijs, Sara; Vries, Sien; van keer, Ines; Arthur-Kelly, Michael; Dind, Juliane; Goldbart, Juliet; Petitpierre, Genevieve; van der Putten, A. A. J.	2021	Looking back, looking forward: Methodological challenges and future directions in research on persons with profound intellectual and multiple disabilities	Journal of Applied Research in Intellectual Disabilities	34	250--262		
48	different topic	Mahoney, Gerald; Robinson, Cordelia; Perales, Frida	2004	Early Motor Intervention - The Need for New Treatment Paradigms	Infants & Young Children	17	291–300		
49	different topic	Marselisborg Centret	2004	Ny indsigt - ny indsats [New insight - new approach]					Arhus
50	different topic	Marsh, James Peter	2005	Coices from a Marginalized Population: Life Histories of Individuals with Physical Impairments					Tampa
51	different topic	McManus, Maura	2007	Intrathecal Baclofen Pumps					
52	different topic	Mensch, S. M.; Echteld, M. A.; Lemmens, R.; Oppewal, A.; Evenhuis, H. M.; Rameckers, E.A.A.	2019	The relationship between motor abilities and quality of life in children with severe multiple disabilities	Journal of Intellectual Disability Research	63	100–112	Wiley-Blackwell	
53	different topic	Mensch, S. M.; Rameckers, E.A.A.; Echteld, M. A.; Evenhuis, H. M.	2015	Reliability of Movakic; an instrument to evaluate motor abilities in children with severe multiple disabilities (PartÔÇÉII)	Physical Medicine and Rehabilitation International	2			
54	different topic	Momola, I.; Szybisty, A.	2011	Intellectually handicapped children vs. Their motor abilities	New Medicine	2011	39–45	Borgis Publishing House	Rzesz
55	different topic	Morgan, Prue; Dobson, Fiona; McGinley, Jennifer	2014	A Systematic Review of the Efficacy of Conservative Interventions on the Gait of Ambulant Adults with Cerebral Palsy	Journal of Developmental & Physical Disabilities	26	633–654	Springer Nature	
56	different topic	Neal, Jerry; Bigby, Linda; Nicholson, Rebecca	2004	Occupational Therapy, Physical Therapy, and Orientation and Mobility Services in Public Schools	Intervention in School & Clinic	39	218–222	Sage Publications	
57	different topic	Nijs, Sara; Vlaskamp, Carla; Maes, Bea	2016	Children with PIMD in interaction with peers with PIMD or siblings	Journal of Intellectual Disability Research	60	28--42		
58	different topic	Nijs, Sara; Vlaskamp, Carla; Maes, Bea	2016	The nature of peer-directed behaviours in children with profound intellectual and multiple disabilities and its relationship with social scaffolding behaviours of the direct support worker	Child: care, health and development	42	98--108		
59	different topic	Paleg, Ginny	2001	Beyond wheelchairs	Rehab Management		60-		
60	different topic	Paleg, Ginny		Moving while standing					
61	different topic	Paleg, Ginny		Up to Speed					
62	different topic	Paleg, Ginny		When needs are special					
63	different topic	Paleg, Ginny; Romness, Mark; Livingstone, Roslyn	2018	Interventions to improve sensory and motor outcomes for young children with central hypotonia: A systematic review	Journal of Pediatric Rehabilitation Medicine	11	57–70	IOS Press	

64	different topic	Pickles, Pilla	2004	Managing the Curriculum for Children with Severe Motor Difficulties: A Practical Approach				David Fulton Publishers	Oxford
65	different topic	Rousseau, M.-C.; Baumstarck, K.; Hamouda, I.; Valkov, M.; Felce, A.; Khaldi-Cherif, S.; Brisse, C.; Loundou, A.; Auquier, P.; Billette de Villemeur, T.; French Polyhandicap Group	2021	Development and initial validation of the polyhandicap severity scale	Revue Neurologique	177	683–689	Elsevier	
66	different topic	Singh, Nirbhay N.	2016	Handbook of Evidence-Based Practices in Intellectual and Developmental Disabilities				Springer International	Cham
67	different topic	Singh, Swarna; Raghumahanti, Raghuveer	2023	Effect of reflex mediated core stabilization and system-based task-oriented approach on motor function and motor ability in children with developmental delay: protocol for a comparative study	F1000Research	12	1534		
68	different topic	Souza de Araujo, Luiz Antonio	2009	Programa de desenvolvimento					Rio de Janeiro
69	different topic	Stamouli, Vasileía	2022	Schediasmós kai ylopoíisi enós mousikou ekpaideftikoú programmatos vasiséno sto programmatos PLAI (Promoting Learning through Active Interaction) gia tin eníschysi tis epikoinonías kai tis allilepídrasis mitéras kai paidiou me varíes í/ kai pollaplés anapirías: mia meléti períptosis. [Design and implementation of a music education program based on the PLAI (Promoting Learning through Active Interaction) program to enhance communication and interaction between mother and child with severe and/or multiple disabilities: a case study]					Thessaloniki
70	different topic	Stasolla, Fabrizio	2021	Rett Syndrome (RTT): from Diagnosis to Treatment				Nova Science Publishers, Incorporated, Nova Science Publishers, Incorporated PP - New York, New York	New York
71	different topic	Strawbridge, Leigh A.	1989	Behavior Therapy Combined with Physical Therapy to Promote Walker Use by a Child with Multiple Handicaps	Education and Training in Mental Retardation	24	239–247		
72	different topic	Turner, Vivian J.	2023	Do we want to add a signature page? Do we want to add values? Mission and vision are included.					
73	different topic	van Alphen, Helena J. M.		Towards evidence-based support in motor activation of people with profound intellectual and multiple disabilities: Chapter 1. General introduction					
74	different topic	van Alphen, Helena J. M.		Towards evidence-based support in motor activation of people with profound intellectual and multiple disabilities: Chapter 3. Development and process evaluation of a motor activity program for people with profound intellectual and multiple disabilities					
75	different topic	van Alphen, Helena J. M.		Towards evidence-based support in motor activation of people with profound intellectual and multiple disabilities: Chapter 4. Construct validity of the Actiwatch-2 for assessing movement in people with profound intellectual and multiple disabilities					
76	different topic	van Alphen, Helena J. M.		Towards evidence-based support in motor activation of people with profound intellectual and multiple disabilities: Chapter 6. General discussion					
77	different topic	van Alphen, Helena J. M.		Towards evidence-based support in motor activation of people with profound intellectual and multiple disabilities: Front matter					

78	different topic	van Alphen, Helena J. M.; Waninge, Aly; Minnaert, Alexander E. M. G.; van der Putten, Annette A. J.	2021	Development and process evaluation of a motor activity program for people with profound intellectual and multiple disabilities	BMC Health Services Research	21	1–13	BioMed Central	
79	different topic	van Alphen, Leentje		Towards evidence-based support in motor activation of people with profound intellectual and multiple disabilities					
80	different topic	van der Ark, L. Andries; Croon, Marcel A.; Sijtsma, Klaas	2008	Possibilities and challenges in Mokken scale analysis using marginal models				Universal Academic Press	
81	different topic	van der Putten, A. A. J.; Vlaskamp, Carla; Luijkx, Jorien; Poppes, Petra; Groningen, Rijkuniversiteit	2017	Kinderen en volwassenen met zeer ernstige verstandelijke en meervoudige beperkingen: tijd voor een nieuw perspectief					
82	different topic	van der Putten, Annette A. J.		Formal handling routines. Child Rearing Practices in Jamaica and Their Relevance to Rehabilitation Work			242–248		
83	different topic	Visser, Linda; Ruiter, Selma A. J.; van der Meulen, Bieuwe F.; Ruijssenaars, Wied A. J. J. M.; Timmerman, Marieke E.	2012	A Review of Standardized Developmental Assessment Instruments for Young Children and Their Applicability for Children With Special Needs	Journal of Cognitive Education & Psychology	11	102–127	Springer Publishing	
84	different topic	Walley, Robert M.	2006	Behavioural phenotypes: approaches to assessment	Assessing Adults with Intellectual Disabilities: A Service Provider's Guide		220	Wiley Online Library	
85	different topic	Waninge, Aly; Rook, R. A.; Dijkhuizen, A.; Gielen, E.; van der Schans, C. P.	2011	Feasibility, test-retest reliability, and interrater reliability of the Modified Ashworth Scale and Modified Tardieu Scale in persons with profound intellectual and multiple disabilities	Research in developmental disabilities	32	613--620		
86	different topic	Ward, Beverly G.	2000	Forging the dream. Proceedings of Symposium IV on African-American Mobility Issues					
87	different topic	Wessels, Marleen D.; van Assen, Arjen A. G.; Post, Wendy J.; van der Putten, Annette A. J.	2023	The construct validity and reliability of the Motor Development List for the assessment of motor skills in children with profound intellectual and multiple disabilities: The next step?	Journal of Intellectual & Developmental Disability	48	370–383		
88	different topic	Wessels, Marleen D.; van der Putten, Annette A. J.; Paap, Muirne C. S.	2021	Inventory of assessment practices in people with profound intellectual and multiple disabilities in three European countries	Journal of Applied Research in Intellectual Disabilities	34	1521–1537	Wiley-Blackwell	
89	different topic	Whinnery, Keith W.		Curriculum Vita					
90	different topic	Whinnery, Stacie B.; Whinnery, Keith W.; Eddins, Daisy	2016	A Strategy for Embedding Functional Motor and Early Numeracy Skill Instruction into Physical Education Activities	Physical Disabilities	35	17–27	Indiana University Press	Arlington
91	different topic	Worms, Lutz; Kemmerling, Manfred	2009	Schwingen auf dem Trampolin: Schwerstbehinderte Menschen erleben ein großartiges Erfahrungsfeld				Meyer & Meyer	London
92	different topic	Young, Courtney; Livingstone, Roslyn; Paleg, Ginny		Team Eakin: Winning the Fight with Treated SMA Type 1					
93	duplicate		2020	Themenschwerpunkt MOVE	heilpädagogik	63	1–30		
94	duplicate	Akiyama, Tomitaro	2003	Some Recent Topics on Cerebral Palsy	The Japanese Journal of Rehabilitation Medicine	40	587–592	The Japanese Association of Rehabilitation Medicine	
95	duplicate	Barnes, Stacie B.	2004	MOVE Program interview with Mom, Steps 1 and 2 [Video]					Bakersfield
96	duplicate	Best, Anthony	2013	Management issues in multiple disabilities				David Fulton Publishers	
97	duplicate	Bidabe, L.	2002	'Do you want to move?'	NEA Today	20	22		
98	duplicate	Bidabe, L.; Lollar, J. M.	1993	Mobility Opportunities Via Education.				verlag modernes lernen	Dortmund
99	duplicate	Bidabe, Linda	1999	MOVE - mobiliy opportunities via education Mobilitätstraining für Kinder und Erwachsene mit Behinderung				Borgmann	Dortmund

100	duplicate	Bidabe, Linda; Lollar, John M.	1990	M.O.V.E. : mobile opportunities via education				Kern County Superintendent of Schools	Bakersfield
101	duplicate	Bidabe, Linda; Voll, C.		No ordinary move				Plough Publishing	Farmington
102	duplicate	Bossink, Leontien	2019	A Move Ahead					
103	duplicate	Brach, Michael	1997	Sport- und Bewegungstherapie in einer stationären Einrichtung für alte Menschen [MOTA – A Mobility Test for Older Adults]	Gesundheitssport und Sporttherapie [Health Sports and Sports Therapy]	13	44–48		
104	duplicate	Brach, Michael; Hasenritter, J.; Kirchner, E.; Bauder-Mißbach, H.; Betschon, E.; Eisenschink, A. M.; Drabner, A.; Panfli, E.	2012	Painful movements and mobility after urological surgery: studying the feasibility of pre-operative exercise	Webmed Central Nursing	3	WMC003102		
105	duplicate	Brown, Fredda; McDonnell, John; Snell, Martha E.	2019	Instruction of Students with Severe Disabilities				Pearson	Upper Saddle River
106	duplicate	Campos, Ana Carolina de; Hidalgo-Robles, Alvaro; Longo, Egmar; Shrader, Claire; Paleg,	2024	F-words and early intervention ingredients for non-ambulant children with cerebral palsy: A scoping review	Developmental medicine and child neurology	66	41--51		
107	duplicate	Campos, Ana Carolina de; Hidalgo-Robles, Alvaro; Longo, Egmar; Shrader, Claire; Paleg,	2024	F-words and early intervention ingredients for non-ambulant children with cerebral palsy: A scoping review	Developmental medicine and child neurology	66	41–51	Blackwell	
108	duplicate	Campos, Ana Carolina de; Hidalgo-Robles, Alvaro; Longo, Egmar; Shrader, Claire; Paleg,	2024	F-Wörter und Interventionsinhalte in der Frühförderung nicht gehfähiger Kinder mit Cerebralparese: eine umfangreiche Literaturübersicht	Developmental medicine and child neurology	66	e23-e34		
109	duplicate	Capone, Kristin; Hoopes, Diana; Kiser, Deborah; Chap	2007	M.O.V.E. (Mobility Opportunities Via Education) Curriculum					
110	duplicate	Capone, Kristin; Hoopes, Diana; Kiser, Deborah; Rolph, Beth	2007	MOVE (Mobility Opportunities Via Education) Curriculum			828–831	Springer	
111	duplicate	Carolina Trombeta Reis; Maria Ines Rubo de Souza Nobre	2014	The professionals' perceptions about the MOVE(r) curriculum approach				Universidade de Sao Paulo	
112	duplicate	Case-Smith, Jane; O'Brien, Jane Clifford	2009	Occupational Therapy for Children				Elsevier	
113	duplicate	Cavanaugh, Lauren K.	2005	Intellectual disabilities		149251 1536			
114	duplicate	Corbeil, Thania	2016	Relever le pari de l´educabilité pur l´elève polyhandicapé: l´émergence d´un accompagnement. [Meeting the Challenge of Educability for the Student with Multiple Disabilities: The Emergence of Support]					Montréal
115	duplicate	Department of Rehabilitation Science and Technology	2007	23rd International Seating Symposium. Moving into the Age of Accountability					
116	duplicate	Ermter, Peggy A.; Glazewski, Krista; Park, SungHee; Haas, Tami; Hudson, Helen; Muehlhausen, Julie; VanHorn, Michelle		A Symposium of PBL-Using Teachers: Teacher Experiences and Student Impact					
117	duplicate	Ikeda, Yoshifumi	2021	Jūdō jūfuku shōgai no aru kodomo no asesumento to kyōiku shien [Assessment and educational support for children with severe and multiple disabilities]	Kyōiku kenkyū jissen hōkoku-shi [Journal of Education Research and Practice]	5	24–33		
118	duplicate	Imray, Peter; Hinchcliffe, Viv	2013	Curricula for teaching children and young people with severe or profound and multiple learning difficulties: Practical strategies for educational professionals				Routledge	Abingdon

119	duplicate	Jansma, Paul	1993	Psychomotor domain training and serious disabilities			University Press of America	Lanham
120	duplicate	Kamenopoulou, Leda	2023	Inclusion education for learners with multi-sensory impairment			Open University Press	Maidenhead
121	duplicate	Kern County Superintendent of Schools	1988	Standing room only; Making strides. [Video]				Bakersfield
122	duplicate	Kern County Superintendent of Schools	2000	Lifting and transferring people with disabilities using the MOVE approach [Video]				Bakersfield
123	duplicate	Kern County Superintendent of Schools	2009	MOVE assessment profile for adults			Kern County Superintendent of Schools	Bakersfield
124	duplicate	Kingsbury, Karen	1991	MOVE Program Makes Inroads in Special Education	Los Angeles Times	1		Los Angeles
125	duplicate	Kuriakidou, Euthymia	2013	Enischysi ton epikoinoniakon symperiforon meso tou programmatos PLAIN se periptosi paidiou me varies h/ kai pollaples anapiries. [Strengthening communication behaviors through the PLAIN program in the case of a child with severe and/or multiple disabilities.]				Thessaloniki
126	duplicate	Kuriakidou, Euthymia	2013	Enischysi ton epikoinoniakon symperiforon meso tou programmatos PLAIN se periptosi paidiou me varies h/ kai pollaples anapiries. [Strengthening communication behaviors through the PLAIN program in the case of a child with severe and/or multiple disabilities.]				Thessaloniki
127	duplicate	Lacey, Penny; Ouvry, Carol	1998	People with Profound and Multiple Learning Disabilities: A Collaborative Approach to Meeting			David Fulton Publishers	Oxford
128	duplicate	Mercieca, Duncan P.	2013	Living Otherwise: Students with Profound and Multiple Learning Disabilities As Agents in Educational Contexts			Birkhauser	Rotterdam
129	duplicate	Miller, Freeman	2005	Cerebral Palsy			Springer	New York
130	duplicate	Miller, Freeman	2007	Therapy, Education, and Other Treatment Modalities	Physical Therapy of Cerebral Palsy	107–136	Springer	
131	duplicate	Miller, Freeman	2007	Cerebral Palsy			Springer	
132	duplicate	Miller, Freeman; Bachrach, Steven; Lennon, Nancy; O'Neil, Margaret E.	2020	Cerebral Palsy			Springer International	Cham
133	duplicate	Miller, Freeman; Peischl, Denise; Koczur, Liz; Strine, Carrie	2007	Seating Systems	Physical Therapy of Cerebral Palsy	361–368	Springer	
134	duplicate	Ministry of Education and Science of the Russian Federation	2016	Pis'mo No. VK-452/07 ot 11 marta 2016 g. O vvedenii FGOS OVZ [Letter No. VK-452/07 dated March 11, 2016. On the introduction of the Federal State Educational Standards for students with special educational needs (SEN)]				Moscow
135	duplicate	Ministry of Education and Science of the Russian Federation	2016	Pis'mo No. VK-452/07 ot 11 marta 2016 g. O vvedenii FGOS OVZ [Letter No. VK-452/07 dated March 11, 2016. On the introduction of the Federal State Educational Standards for students with special educational needs (SEN)]				Moscow
136	duplicate	Ministry of Education and Science of the Russian Federation	2016	Pis'mo No. VK-452/07 ot 11 marta 2016 g. O vvedenii FGOS OVZ [Letter No. VK-452/07 dated March 11, 2016. On the introduction of the Federal State Educational Standards for students with special educational needs (SEN)]				Moscow
137	duplicate	Ministry of Education and Science of the Russian Federation; Ministry of Education and Science of the Russian Federation		Methodological recommendations on the implementation of the federal state educational standard for the initial general education of students with disabilities and the federal state educational standard for the education of students with mental retardation (intellectual disabilities)				
138	duplicate	Nakken, Han	2005	Targets halen in het onderzoek naar problemen bij het opvoeden van kinderen die in hun ontwikkeling worden belemmerd. [Setting targets in research on problems in raising children who are hindered in their development]				

139	duplicate	Nalbant, Sibel	2011	Haftalık fiziksel aktivite programının Down Sendromlu çocukların motor gelişimleri ve günlük yaşam aktiviteleri üzerindeki etkisinin incelenmesi. [14-week physical activity programme for motor development and activities of daily living in children with down syndrome]					Ankara
140	duplicate	Ott, Donna DeSanto; Effgen, Susan K.	2000	Occurence of Gross Motor Behaviors in Integrated and Segregated Preschool Classrooms	Pediatric physical therapy	12	164–172		
141	duplicate	Paleg, Ginny; Livingstone, Roslyn	2015	Outcomes of gait trainer use in home and school settings for children with motor impairments: a systematic review	Clinical rehabilitation	29	1077–1091		
142	duplicate	Pyo, Yun H.	2011	Jeonmun-ga gan-ui hyeobryeokjeok team jeopgeun joenja yeongchal jangae yeongyu-a-reul wihan gyosa wa gwallyeon seobiseu munga gan-ui hyeobryeokjeok team jeopgeun joenja yeongchal. [A Review on Research regarding Collaborative Team Approach Intervention for the Young Children with Disabilities]	Teuksu Gyoyuk [Spec Educ Res]	10	55–77		
143	duplicate	Ramos, Marisol	2013	A project proposal for designing a management information system to report the effectiveness of the MOVE program					Bakersfield
144	duplicate	Reis, Carolina Trombeta; Nobre, Maria In├s Rubo de Souza	2014	Las percepciones de los profesionales sobre el abordaje del programa MOVE (r)	Fisioterapia e Pesquisa	21	133–138	SciELO	
145	duplicate	Rennie, Jeanette	2009	Learning Disability: Physical Therapy Treatment and Management, a Collaborative Appoach				John Wiley & Sons	
146	duplicate	Schomerus, Riclef	1996	Written term paper as part of the First State Examination for the teaching profession for special education					Dortmund
147	duplicate	The Disability Partnership	08/03/2000	Moving towards inclusion. MOVE conference proceedings				The Disability Partnership	London
148	duplicate	Toyama, Tomatiro Akiyama	2003	Nōsei mahi no saikin no wadai	Rihabiriteshon Igaku [Nippon Rehabil Med]	40	587–592		
149	duplicate	van der Meulen, B.; Vlaskamp, Carla; van den Bos, K. P.	2005	Interventies in de orthopedagogiek [Interventions in special needs education]				Lemniscaat	Rotterdam
150	duplicate	Whinnery, Keith W.; Barnes, Stacie B.	2002	Mobility training the MOVE (R) curriculum	TEACHING Exceptional Children	34	44–50		
151	duplicate	Young, Beverly	2008	A Collaborative Effort Allows People with Disabilities to Experience the Joy of Horseback Riding	Exceptional Parent	38	33		
152	duplicate	Zoltan, Lenart	2019	Spasztikus kus cerebralis paretikus tanulok fels vegtagi mozgasainak fejese egy tanev alatt: Vizsgeati lehetegek pedagogiai szi}nteren es egyes merhet valtozasok					Budapest
153	internet only	Levin, Deborah J.	2012	Seating and Mobility in School Aged Children with Special Needs				Anthem Media Group	Los Angeles
154	introduction	Logan, Jim		Chairman of the International Research Conference			2		
155	introduction	Penn, Claudia	2000	MOVE in der Inklusion	heilpädagogik				
156	introduction	Penn, Claudia		Vernetzung von Pädagogik und Physiotherapie					
157	not retrievable		1993	Making his MOVE	Orlando Sentinel		-I6		Orlando
158	not retrievable		1999	Your future: University supports charity for disabled	Evening Mail		65		Birmingham
159	not retrievable		2001	Challenge Day Games	The Washington Post				
160	not retrievable		2001	SCHOOL NOTES	The Washington Post		-T09		Washington D.C.
161	not retrievable		2002	Programme helps get students up and about	Evening Post		20		Wellington
162	not retrievable		2006	Santa Rosa School Briefs	Pensacola News Journal				Pensacola
163	not retrievable		2006	UNIVERSITY OF WEST FLORIDA TO HOST 'EVENING WITH FRIENDS OF MOBILITY OPPORTUNITIES VIA EDUCATION'					Washington D.C.
164	not retrievable	Alicia Di Rado	1992	Helping Young Felons by Letting Them Help Others	Los Angeles Times		1		Los Angeles
165	not retrievable	Anderson, Jessie; Manning, Stephen	1	Two steps forward, none back	Times Educational Supplement		56–57		
166	not retrievable	Basler, George	2011	IMPROVING INDEPENDENCE-MOVE program improves mobility of BOCES students: Forum introduces efforts to agencies that work with disabled	Press & Sun-Bulletin				Binghamton
167	not retrievable	Basler, George	2011	MOVE program improves mobility of BOCES students	Press & Sun-Bulletin				Binghamton

168	not retrievable	Becker, Stacey	2012	Eisenhower runs with mobility program	Telegraph - Herald				Dubuque
169	not retrievable	Becker, Stacey	2013	Experience MOVEs students	Telegraph - Herald				Dubuque
170	not retrievable	Castellano, Carol	2005	Making It Work: Educating the Blind/Visually Impaired Student in the Regular School				Information Age Publishing	Charlotte
171	not retrievable	Cavanaugh, Patricia E.		Disability Awareness Nights 2004	Exceptional Parent	34	50–54		
172	not retrievable	Clark-Boll, Rebecca Diane	2002	School-Based Occupational Therapists' Reported Frames of Reference, Therapeutic Approaches, Assessment Practices, and Goals for Students with Moderate to Severe Disabilities					Albany
173	not retrievable	Clark-Boll, Rebecca Diane	2002	School-based occupational therapists' reported frames of reference, therapeutic approaches,* assessment practices, and goals for students with moderate to severe disabilities				State University of New York at Albany	
174	not retrievable	Davidson, Kathleen M.		New Zealanders on MOVE : perspectives of its effectiveness: a case study					
175	not retrievable	Dizon, Lily	1993	Young Offenders Are Able to Help County Wards Working With Disabled Children See What Real Misfortune Is, Learn Joy of Giving	Los Angeles Times		1		
176	not retrievable	Farrell, Michael	2016	Educating Special Students: An Introduction to Provision for Learners with Disabilities and Disorders				Taylor & Francis	Oxford
177	not retrievable	Freiberg, Karen L.	2005	Educating exceptional children 05/06				McGraw-Hill	Dubuque
178	not retrievable	Goodwin, Jamie	2003	Program improves students' health: New therapy helps kids with disabilities at 10 Westside schools develop motor skills	Indianapolis Star				
179	not retrievable	Henderson, Maurice	1993	Play about MOVE should be praised	Philadelphia Tribune		1		Philadelphia
180	not retrievable	Hillary Whitcomb Jesse	2005	Woodland program to be national model	Times Herald				Port Huron
181	not retrievable	Hoey, Kim	2011	Team helps put South Africans on their feet	The News Journal				Wilmington
182	not retrievable	Jarvis, Fred	1997	Life on the Move: Fred Jarvis looks at a new approach to coping with disability	The Guardian		1		London
183	not retrievable	Jessee, Ronald		Effects of Functional Mobility Skills Training for Young Students With Physical Disabilities					
184	not retrievable	Kaganov, Veniamin	2016						
185	not retrievable	Kelly, Paula F.	2005	On a path to better health and enriched lives: Program helps disabled develop motivation to be mobile					Wilmington
186	not retrievable	Kern County Superintendent of Schools	1988	Making strides: an update of Standing room only [Video]					Bakersfield
187	not retrievable	Kern County Superintendent of Schools	2006	Freedom to MOVE [Video]				Kern County Superintendent of Schools	Bakersfield
188	not retrievable	Kushvakha, N. G.		Osobennosti diagnostiki, otsenki dvigatel'nykh vozmozhnostey i potrebnostey, podbor TSR i vklyuchenie trebuemogo razvitiya dvigatel'nykh navykov v programmy reabilitatsii pri vyrazhennoy dvigatel'noy patologii u detey raznogo vozrasta. [Features of assessment, evaluation of motor abilities and needs, selection of assistive devices, and incorporation of required motor skill development into rehabilitation programs for children of different ages with severe motor impairments.]					
189	not retrievable	LaRosa, Vanetta R.	05/2007	Validation of preference assessment involving persons with varying degrees of multiple disabilities through contingent and non-contingent stimulus use in daily activity routines					Carbondale
190	not retrievable	Lauteslager, Peter; van der Burgt, Ineke; van den Heuvel, Marieke; van der Sien, Ria Nijhuis; van der Putten, A. A. J.		Kinderen met syndromen en verstandelijke beperkingen				Springer	
191	not retrievable	Lunnen, K. Y.; Geddes, R. F.	2014	Physical therapy in the educational environment			717–734		
192	not retrievable	Maeshiro, Karen	2000	Big Wheels on Campus. Disabled Students gain mobility: [Antelope Valley Edition]	Daily News		-AV1		Los Angeles

193	not retrievable	Maison, Diana	2011	MOVE makes a difference at Woodland	Times Herald				Port Huron
194	not retrievable	Mann, Laura	2007	ON THE MOVE	Newsday				Long Island
195	not retrievable	Miller, Beth	2007	Program gets disabled on the MOVE	The News Journal				Wilmington
196	not retrievable	Minchau, Andreas		M.O.V.E. (mobility opportunities via education) mit Menschen mit geistiger Behinderung Möglichkeiten und Grenzen					
197	not retrievable	Ministry of Education and Science of the Russian Federation		How to manage inclusive classroom effect					
198	not retrievable	Pierce, Harold	2017	Linda Bidabe, founder of global nonprofit that helps developmentally disabled students gain mobility, dies at 72	TCA Regional News				Chicago
199	not retrievable	Sanchez, Edgar	2007	Kids on the move: Special program improves motor skills for youngsters with disabilities	Tribune Business News				Washington
200	not retrievable	Schack, T.; Pollmann, D.	2020	Promotion of development of childhood motor function: Motor diagnostics and interventional concepts in kindergarten and elementary school age	Monatsschrift fur Kinderheilkunde	168	215–221	Springer Medizin	
201	not retrievable	Sobek, Stephen	1999	Tiny steps, gigantic courage	The News Journal				Wilmington
202	not retrievable	Stanton, Marion	2012	Understanding Cerebral Palsy: A Guide for Parents and Professionals				Jessica Kingsley Publishers	London
203	not retrievable	Tada, Wendy L.; Harris, Susan R.	1986	Physical Therapy in the Educational Environment					
204	not retrievable	Talalay, Sarah	1996	Program for disabled gets dreams moving	Sun Sentinel				Fort Lauderdale
205	not retrievable	Truett, Richard	1993	M.O.V.E. kids don't have time to just sit around	Orlando Sentinel		-I1		Orlando
206	not retrievable	van der Putten, A. A. J.; Nakken, H.	2004	Verbeteren van de zorg voor kinderen met ernstige meervoudige beperkingen door middel van het MOVE curriculum				Stichting Kinderstudies	
207	not retrievable	van Empelen, Ron; Nijhuis-van der Sanden, Ria; Hartman, Annelies	2017	Kinderfysiotherapie				Bohn Stafleu en van Loghum	Houten
208	not retrievable	Vasileia, Stamouli		Didomatik Ergasia?					
209	not retrievable	Vuijk, P. J.; Hartman, E.; Scherder, E.; Visscher, C.	2010	Motor performance of children with mild intellectual disability and borderline intellectual functioning	Journal of Intellectual Disability Research	54	955--965		
210	not retrievable	Zervas, Demitris		000-videnscenter					
211	presentation	Sues-Delaney, Julie; Harrison, Melinda		MOVE Program. Mobility Opportunities via Education (R) / Experience [Presentation]					
212	wrong reference	Tada, Wendy L.; Harris, Susan R.	1986	Therapeutic Exercise in Developmental Disabilities				Aspen Systems	Rockville
