# Supplementary material for: Promoting Functional Mobility in Individuals with Non-Ambulatory Cerebral Palsy: A Scoping Review of the MOVE Programme
Source: Children (Basel). 2026 Feb 20;13(2):292. doi: 10.3390/children13020292 (PMC12939002; doi:10.3390/children13020292)
Supplement: Supplementary file 1 [file children-13-00292-s001.zip › Schomerus supp table S3 included literature.pdf]

Citation Number	Author	Year	Categories	Content	Type of publication	Language	Geographic location	Relationship to MOVE
13	van der Putten, Annette A. J.	2017	4 Concept	Motor activation plays a minor role in the support for people with PIMD; reasons: assumption that these people cannot move or do not profit from movement-oriented activities; lack of knowledge about the development of motor functioning; lack of evidence-based interventions - only MOVE and active support known to the authors	journal article	English	Netherlands	one of several topics
17	Baumgart, Diane; Brown, Lou; Pumpian, Ian; Nisbet, Jan; Ford, Alison; Sweet, Mark; Messina, Rosalie; Schroeder, Jack	1982	conceptual foundation	partial participation supports appropriate education for students with severe disabilities	*not considered*	*not considered*	*not considered*	*not considered*
18	Bax, Martin	1986	conceptual foundation	abnormal reflexes,' 'primitive reactions' and muscle tone are an adaptation of the nervous system; students must learn to move under these conditions	*not considered*	*not considered*	*not considered*	*not considered*
19	Bleck, Eugene E.; Nagel, Donald A.	1982	conceptual foundation	non-ambulatory students over the age of seven usually do not progress in their motor skills; weight bearing helpful for hip health	*not considered*	*not considered*	*not considered*	*not considered*
20	Bobath, Karel; Bobath, Berta	1984	conceptual foundation	approach has moved from focussing on body structure and function (e. g. reflexes) to more functional focus	*not considered*	*not considered*	*not considered*	*not considered*
21	Brown, Lou; Branston, Mary Beth; Hamre-Nietupski, Susan; Pumpian, Ian; Certo, Nick; Gruenewald, Lee	1979	conceptual foundation	outlines the need for age-appropriate goals and functional curricula for students with severe disabilities	*not considered*	*not considered*	*not considered*	*not considered*
22	Campbell, Philippa H.	1987	conceptual foundation	physical management within Snells framework; emphasises postural management and tone	*not considered*	*not considered*	*not considered*	*not considered*
23	Campbell, Philippa H.	1987	conceptual foundation	tone regulation important to teach motor functions	*not considered*	*not considered*	*not considered*	*not considered*
24	Campbell, Philippa H.; McInerney, William F.; Cooper, Margaret A.	1984	conceptual foundation	age-appropriate, functional goals should guide physiotherapy for students with severe disabilities; assessment should therefore focus on function	*not considered*	*not considered*	*not considered*	*not considered*
25	Mulcahy, C. M.; Pountney, T. E.; Nelham, R. L.; Green, E. M.; Billington, G. D.	1988	conceptual foundation	upright sitting improves perception and motor function, compared to reclined sitting; proposes a functional adaptive chair	*not considered*	*not considered*	*not considered*	*not considered*
26	Scrutton, David	1984	conceptual foundation	scoliosis follows misalignment of the hips in sitting	*not considered*	*not considered*	*not considered*	*not considered*
27	Snell, Martha E.	1987	conceptual foundation	proposes a structured approach for individual appropriate education of students with severe disabilities; steps similar to the MOVE programme; obviously a source for the structure of the MOVE programme	*not considered*	*not considered*	*not considered*	*not considered*
28	Barnes, Stacie B.	1997	Case Study	Six case studies of elementary students	video	English	USA	main topic
29	Barnes, Stacie B.	1999	Theory; Study	critiques developmental approaches, which diminish the opportunities to practice functional tasks and which lead to segregation from peers, and which are discontinued due to slow progress, resulting in demobilisation.<br>Theoretical description (McGraw, Gesell; NDT, SI; Dynamical Systems Theory, neuronal group selection; support model; Vygotski - support fading out; integrated therapy; brief description of the MOVE Curriculum (top down approach, six steps) MOVE development (time, rate of learning, generalization); pilot project; alignment with contemporary Theory.<br>Study on the effectivity of MOVE (but social skills now not addressed anymore): 5 children, 3-9y, multiple baseline across-subjects design;<br>Study results and discussion: support MOVE, variability in data<br>Limitations: small sample size, generalisability<br>future research: direct replication - reliability; systematic replication - generalisability	dissertation	English	USA	main topic
30	Barnes, Stacie B.; Whinnery, Keith W.	2000	Theory; Study;	MOVE conference presentation of concept and background/Theory and current research	conference abstract	English	USA	main topic
31	Barnes, Stacie B.; Whinnery, Keith W.	2002	Study	summary of dissertation	journal article	English	USA	main topic
32	Bidabe, D Linda	1993	History/Development; Concept; Equipment; Assessment; Case reports; Implementation	short description of the programme and anecdotal evidence from practical work with 200 students	book chapter	English	USA	main topic
33	Bidabe, D Linda	1997	History/Development; Concept; Equipment; Assessment; Study reporting	answers common questions about MOVE, Top-Down vs. Bottom-Up; Motivation to develop MOVE, Time/Rate of Learning/Generalization; six steps; research, equipment, physical education	book chapter	English	USA	main topic

34	Bidabe, D Linda; Lollar, John M.	1990	History/Development; Concept; Equipment; Assessment; Case reports; Implementation	describes the six steps of the MOVE program in detail, case examples	book	English	USA	main topic
35	Bidabe, D Linda; Lollar, John M.	1993	History/Development; Concept; Equipment; Assessment; Case reports; Implementation	German translation of Bidabe and Lollar 1990	book	German	USA	main topic
36	Bidabe, D Linda; Lollar, John M.	1994	History/Development; Concept; Equipment; Assessment; Case reports; Implementation	Spanish translation of Bidabe and Lollar 1990	book	Spanish	USA	main topic
37	Bidabe, D Linda; Martin, M.; Yates, Robyn; Binns, Chris	1988	History/Development; Concept; Equipment; Study	pilot study during the development of the MOVE programme; working title "standing room only"; case studies on the influence of equipment on functional movement; description of the equipment; mentions literature that MOVE is based upon;	journal article	English	Australia	main topic
38	Bidabe, D Linda; Voll, Chris	2001	History/Development; Equipment; Case reports	autobiography of Linda Bidabe, describes the experiences in her work that lead to the development of the MOVE programme as well as the development itself	book	English	USA	one of several topics
39	Bossink, Leontien	2019	Study reporting	discusses physical activity of people with profound intellectual and multiple disabilities, mentions MOVE once	dissertation	English	Netherlands	one of several topics
40	Brach, Michael; Schomerus, Riclef	2000	Study reporting	compares the different approaches of research using a research framework	conference abstract	English	Germany	main topic
41	Case-Smith, Jane; O'Brien, Jane Clifford	2014	Concept; Study reporting	short description of MOVE; motor control and task-specific approach; child-centered goals; some effectivity shown in studies	book	English	USA	
42	De Campos, Ana Carolina; Hidalgo-Robles, Álvaro; Longo, Egmar; Shrader, Claire; Paleg, Ginny	2024	Study reporting	assigns interventions for children at high risk to become non-ambulatory to the f-words. Mentions the Whinnery study in fitness.	journal article	English	Brazil	one of several topics
43	De Campos, Ana Carolina; Hidalgo-Robles, Álvaro; Longo, Egmar; Shrader, Claire; Paleg, Ginny	2024	Study reporting	Portuguese translation of De Campos et al. 2024	journal article	Portuguese	Brazil	one of several topics
44	De Campos, Ana Carolina; Hidalgo-Robles, Álvaro; Longo, Egmar; Shrader, Claire; Paleg, Ginny	2024	Study reporting	Spanish translation of De Campos et al. 2024	journal article	Spanish	Brazil	one of several topics
45	De Campos, Ana Carolina; Hidalgo-Robles, Álvaro; Longo, Egmar; Shrader, Claire; Paleg, Ginny	2024	Study reporting	German translation of De Campos et al. 2024	journal article	German	Brazil	one of several topics
46	Elkins, Kathleen M.	1994	History/Development; Concept; Theory; Study	Comprehensive overview over history and development of special education preparing the development of the MOVE Curriculum: insufficient school and therapy situation PMLD, developmental model, MOVE Curriculum; Study: comparison MOVE and traditional models; purpose: sound curricula; inclusion in developing special education settings; history of special ed for PMLD in the USA; expecting too little from PMLD students; new, inclusive Curriculum for PMLD students;<br>history of curricula for PMLD; developmental approach, not age appropriate; -> functional curricula<br>critique of Piaget, SI and NDT<br>Study on the effectiveness of MOVE with younger and older students, assessed with the TDMMT, in comparison to usual care. result: very effective	dissertation	English	USA	main topic
47	Gaiatto, Elisabetta	2000	Case reports; Implementation	pilot study with 55 severely disabled patients in a hospital; significant improvement; influence on surgery decisions;	conference abstract	English	Italy	main topic
48	Galdin, Marlène; Robitaille, Luc; Dugas, Claude	2010	Study reporting	Review of techniques and programs to increase functional motor skills in adolescents and adults with multiple disabilities; four intervention strategies: therapeutic, bottom-up, top-down, new technologies; top-down approaches and new technologies seem to have positive effects, other than bottom-up, sensory focussed approaches like snoezelen, Multisensory Environments; no studies for comparison between bottom-up and top-down approaches;	journal article	French	France	one of several topics
49	Garrett, Kristi	2012	Case reports; Implementation	development of MOVE; 25 countries, 13 languages; "can do approach", change in expectations what pupils can achieve	periodical	English	USA	main topic
50	Gebhard, Britta	2005	History/Development; Concept; Study reporting	describes the concept, history of MOVE	book chapter	German	Germany	one of several topics

51	Gonzales, Jennifer J.; Caballero, Vicente A. G.	2022	Study	MOVE case series with adults in a day care center; using TDMMT and Prompt Reduction Plan as assessments; PRP - likert scale significant improvements - but methodological problems (assessments); Hebrew with additional English abstract	journal article	Hebrew	Israel	main topic
52	Graff, Berthold	1996	Concept; Study; Implementation	Case-study with four students; one year of MOVE training; progress in motor and other domains	master thesis	German	Germany	main topic
53	Haigh, Gerald	1991	Case reports; Implementation	plain language description; teaching mobility instead of following developing sequences; success stories from one school	news article	English	UK	main topic
54	Houwen, Suzanne; van der Putten, Annette A. J.	2014	Study reporting	Evidence: motor intereventions designed to improve motor/cognitive/social outcomees; review, study of van der Putten; effects of motor interventions on cognitive and social functioning remain unknown (van der Putten, 2005)	journal article	English	Netherlands	one of several topics
55	Houwen, Suzanne; van der Putten, Annette A. J.; Vlaskamp, Carla	2015	Study reporting	dutch version of Houwen and van der Putten 2014	journal article	Dutch	Netherlands	one of several topics
56	Jean, Shirley D.	2020	Study; Implementation	Qualitative study on the socio-emotional growth, functional mobility, progress and attitudes of the school site staff; based on "transformative learning" (Mezirow); detailed insights into the perspectives of 14 (?) MOVE practitioners/trainers.<br>Results of the study could be included into the ScR.<br>My feeling is that it is not a high quality dissertation though (references, methodology, coherence).	dissertation	English	USA	main topic
57	Kern County Superintendent of Schools	2006	History/Development; Equipment; Case reports; Implementation	History of MOVE, development and trial of MOVE adults; development of the toileting program within MOVE for adults	video	English	USA	main topic
58	Kern County Superintendent of Schools	2008	Case reports; Implementation	Explanation of MOVE for adults; canges for the service for adults with disabilities, no longer overlooked; three case stories; and for the institution/staff/team; influence on health and alertness; participation; emotion; behaviour	video	English	USA	main topic
59	Kern County Superintendent of Schools	2009	Derivatives	In depth explanation of the Hygiene and toileting program; All mobility skills can be practiced in the toileting situation: sitting, standing, walking, transitioning; Overview of the four levels; How to implement the six steps; Step 1 - interview and Top Down Toileting Assessment; Step 2 - Establish a team; select basic routine for individual learner (Instructions for selecting); Step 3 - Select and modify equipment and surrounding; finish Top Down Toileting Assessment; Step 4 - target critical skills (4 sections: sitting skills; transition and movement skills; hand and arm use skills; communication skills); Step 5 - develop individualized plans for improving skills; teaching guidelines in the program; Step 6: teach and generalize the skills: generalize learner's skills; teaching support providers;	video	English	USA	main topic
60	Kern County Superintendent of Schools	2009	Equipment; Case reports; Derivatives	Level III Basic Toileting Routine; Case story Kelly	video	English	USA	main topic
61	Kern County Superintendent of Schools	2009	Equipment; Case reports; Derivatives	Level I Basic Toileting Routine; Case story Marlen	video	English	USA	main topic
62	Kern County Superintendent of Schools	2010	History/Development; Equipment; Study reporting; Case reports; Derivatives	MOVE for adults started in 2003; description of the concept - six steps: 1 MOVE assessment (1-Learner profile interview - what is the learners life situation? 2-TDMMT); 2 Setting goals; 3 Planning activities (task analysis); 4 measuring prompts; 5 prompt review; 6 teaching skills	book	English	USA	main topic
63	Kern County Superintendent of Schools	n.d.	Case reports; Implementation	testimonials of a mother, grandfather and teacher of one boy with disabilities	video	English	USA	main topic
64	Kingsbury, Karen	1991	History/Development	Story of Tommy, who got the first gait trainer in 1978 and died later after being transferred to another school, where he was immobilised. Starting point for Bidabe to start MOVE. Development of MOVE contradictory to traditional thinking; other case stories with parents views.	news article	English	USA	main topic
65	Kozleski, Lisa	2001	History/Development; Case reports; Implementation	Case stories of several students from one school, with quotes from their parents.	news article	English	USA	main topic
66	Kuriakidou, Euthymia	2013	Concept; Study reporting	very short description of MOVE, conveys van der Putten's 2004 study on the goal-analysis	master thesis	Greek	Greece	one of several topics

67	Lehoux, Marie-Claude	2018	Study reporting	report on programs for adults with multiple handicaps for the improvement of services in Quebec/Canada; the clients are passive; no evidence basedpractice guidelines; question: what interventions should be implemented?; effects on QoL; quite general recommendations for practice; more research on effectivity needed	grey literature	French	France	one of several topics
68	Levy, Rebecca	2023	Study;	Study protocol, whether participation in MOVE is associated with reduced use of healthcare for individuals with developmental disabilities, unclear status of the study	protocol	English	USA	main topic
69	Livingstone, Roslyn; Paleg, Ginny	2016	Equipment; Assessment; Study reporting	Review of assessments related to assisted walking; mentions TDMMT;	journal article	English	Canada	one of several topics
70	Livingstone, Roslyn; Paleg, Ginny	2023	Equipment; Study reporting	Reports studies by van der Putten in the context of supported stepping devices	journal article	English	Canada	one of several topics
71	Low, Sheryl A.; McCoy, S. W.; Beling, J.; Adams, J.	2005	Study	Pilot study on effectiveness of MOVE: 39 children 3.5-13 years, median 9.2 years, Range of Motion, Revised Gesell Developmental Schedule, TDMMA, pre-post, no control group, 79% gains, 8% no changes, 13% lost function in functional mobility; ROM: 30%+, 57%x, 13%-; Developmental level: 28%+, 69%x, 3%-; decreases partly by medical conditions; one independent walker; younger as well as older students made progress; increased alertness, ease of care reported	journal article	English	USA	main topic
72	Lucey, Dorothy; Goen, Ben	nd	Case reports; Implementation	TV-show on the MOVE-program with help by juvenile offenders; testimonials by the parents of the disabled children and the adolescents; both groups make steps to independence	TV show	English	USA	main topic
73	Maes, Bea	2007	Study reporting	Review of interventions for PIMD; life situation of individuals with PIMD; moderate effect of MOVE on independence when performing movement skills (Van der Putten et al. 2005)	journal article	English	Belgium	one of several topics
74	Mensch, Sonja M.	2017	Study reporting; Derivatives	existing assessments did not fit the criteria for clinical use with individuals with complex disabilities (low difficulty, grading of scores, support, non-verbal, capability); new instrument was developed (MOVAKIC)	dissertation	English	Netherlands	one of several topics
75	Mensch, Sonja M.; Rameckers, E. A. A.; van den Boogaard, P.; Ketelaar, Marjolijn	2005	Assessment; Study reporting; Derivatives	literature search of measures of changes in motor skills of children with complex disabilities; six assessments found; requirements: low level of difficulty and small steps; no test reaches the requirements, resulting in a need for a new assessment; in the TDMMT, the steps are too big	journal article	Dutch	Netherlands	one of several topics
76	Mensch, Sonja M.; Rameckers, E. A. A.; Echteld, Michael A.; Evenhuis, Heleen M.	2015	Assessment; Study reporting; Derivatives	existing assessments did not fit the criteria for clinical use with individuals with complex disabilities (low difficulty, grading of scores, support, non-verbal, capability); new instrument was developed (MOVAKIC)	journal article	English	Netherlands	one of several topics
77	Mercieca, Duncan P.	2013	Case reports; Brief mentions	case story of a student with severe physical disabilities and secondary impairments, who is very vulnerable and difficult to position; mentions a teacher visiting from England once a year for several weeks, trained in the MOVE program	book	English	Malta	brief mention
78	MOVE - Bewegung fürs Leben	2024	History/Development; Concept; Equipment; Assessment; Study reporting; Case reports; Implementation	Background and comprehensive information on implementation; official resource in Austria and Germany	book	German	Austria	main topic
79	MOVE Austria	2019	History/Development; Concept; Equipment; Assessment; Study reporting; Case reports; Implementation	Official trainer's material for Germany and Austria; Background information, training guidelines	book	German	Austria	main topic
80	Nakken, Han; Reynders, Koop; Vlaskamp, Carla; Procee, A. I.	1998	History/Development; Concept; Equipment; Study reporting; Implementation	compares eleven interventions for severely disabled, severel categories by parents, pracitioners, scientists; MOVE a bit positive	book	Dutch	Netherlands	one of several topics
81	Paleg, Ginny	1997	Case reports; Implementation	case report Michael, 5 years, athetotic CP, MOVE since 12 month of age; learned to sit independently, pull to stand, walk with gait trainer, communicate, interact and feed himself.<br>Case report by mother of Michael, 17, who learned to make his first steps in a gait trainer and began to speak	periodical	English	USA	main topic
82	Paleg, Ginny	1997	Case reports; Implementation	case study of four years of MOVE progress in a boy with severe disabilities; anecdotal success including functional (body) improvements (less scoliosis, contractures, hip dislocation);	periodical	English	USA	main topic

83	Paleg, Ginny	1997	Study	retrospective descriptive case series; in-patient pediatric rehabilitation; 6 females, 13 males, mean age 6 (2-14) with non-ambulatory CP; one week intensive in-patient stay, 20 hours of therapy; TDMMT on day one and day five; scored as dependent or independent in sitting, standing and walking; 18/19 learned to walk, 11 to sit, 9 to stand; provides evidence for the effectivity of MOVE; challenge developmental approaches; walking was easier to learn than standing and sitting;	journal article	English	USA	main topic
84	Paleg, Ginny; Livingstone, Roslyn	2015	Study reporting	review of studies on gait trainer use; including Barnes, Whinnery, van der Putten studies; positive outcomes of gait trainer use within the MOVE programme on activity domain of the ICF	journal article	English	USA	one of several topics
85	Penn, Claudia	2020	Concept; Case reports; Implementation	Role of the family: feel recognised with their demands and wishes; quotes from families	periodical	German	Austria	main topic
86	Penn, Claudia	2020	Concept; Case reports	Case report Tommi by his mother; basic ideas of MOVE	periodical	German	Austria	main topic
87	Ramos, Marisol	2013	Study;	nonprofit organisations collect outcome data for fundraising reasons, to demonstrate the effectivity of their interventions; literature research on outcome report models; detailed description of the MOVE concept, closely follows the usual description; outcome report would use TDMMT results as outcomes; costs of almost 200.000$/year; cooperation with university could save costs	master thesis	English	USA	main topic
88	Reis, Carolina Trombeta	2011	Study	Dissertation on the use of MOVE in Brazil	dissertation	Portuguese	Brazil	main topic
89	Reis, Carolina Trombeta; Rubo de Souza Nobre, MI	2014	Study	perception of teachers and assistants in Brasil towards the MOVE Curriculum; 17 semi-structured interviews; humanized view of children; active role of assistants, teamwork; motivation of the child while using MOVE; importance of motor learning in natural settings	journal article	Portuguese	Brazil	main topic
90	Reis, Carolina Trombeta; Rubo de Souza Nobre, MI	2014	Study	qualitative case study on the perceptions of the teachers and assistants regarding MOVE; 17 semi-structured interviews; team approach, humanization, motivation of the students, motor learning in everyday life to achieve functionality and independence	conference abstract	English	Brazil	main topic
91	Reis, Carolina Trombeta; Rubo de Souza Nobre, MI	2014	Study	perception of teachers and assistants in Brasil towards the MOVE Curriculum; 17 semi-structured interviews; humanized view of children; active role of assistants, teamwork; motivation of the child while using MOVE; importance of motor learning in natural settings	journal article	English	Brazil	main topic
92	Reynders, Koop	2005	Theory; Study reporting	Children with PIMD/EEI are often inactive, activity is necessary for learning and development (neurophysiology, Edelman, Hebb); programs to activate children with PIMD: MOVE, but needs theoretical underpinning and implementation strategy.	book chapter	Dutch	Netherlands	one of several topics
93	Roseman, Eileen	2005	Case reports	descriptive case story of one adolescent who began with MOVE at the age of 14 and got independent of his wheelchair at the time of his graduation	periodical	English	USA	main topic
94	Sarimski, Klaus	2021	Concept; Study reporting	general about motor abilities	book	German	Germany	one of several topics
95	Schomerus, Riclef	1996	Concept; Theory; Study	adaptational motor development, MOVE concept; single case study, diverse improvements, changes in attitudes of the teachers	master thesis	German	Germany	main topic
96	Schomerus, Riclef	1998	Concept; Theory; Study	essence of the master thesis	journal article	German	Germany	main topic
97	Segal, Aina	1998	Case reports; Brief mentions	brief mention: School uses MOVE alongside therapeutic horseback riding	dissertation	English	UK	brief mention
98	Tedla, Jaya S.; Ganesan, Sailakshmi; Katregadda, Srinadh	2009	Assessment; Study	psychomotor analysis of the TDMMT	journal article	English	India	main topic
99	Thomson, Gilbert	2005	History/Development; Concept; Equipment; Theory; Case reports	most comprehesive theoretical background - disability, motor control, motor development, motor learning, task-oriented intervention	book	English	USA	main topic
100	Trepanier, Anne Marie	2008	Study	Description of MOVE, MOVE for adults, need for motor activation in adults with disabilities; is a program with MOVE components effective? is the TDMMT effective in evaluating functional skills? pilot study with 6 adults, single-subject multiple baseline, 8 (7) weeks, TDMMT pre-posttest; five/six participants (partially) reached their goal within the eight weeks; decline in self-injuring behaviour in one participant; not exactly clear, shich components of the MOVE program were used!!	grey literature	English	USA	main topic

101	van der Putten, Annette A. J.	2000	Study	Outlines the study: content analysis - if MOVE works and how MOVE works: Intervention, Context, Outcome, Intervening mechanisms; Methodology and preliminary outcomes, two illustrative case reports. Goal: find a Theory that explains the effects of the MOVE programme	conference abstract	English	Netherlands	main topic
102	van der Putten, Annette A. J.	2005	Assessment; Study; Derivatives	psychometric analysis of the TDMMT; shift to functional interventions needs functional assessments, description of the TDMMT (unclear, what mastering an item means - reliability!!), methodology: test-retest and inter-rater reliability - PT and OT scored three boys twice; analysis of ordering of the items - all items were tested; validity: RESULTS: Factor analysis - not 3 factors (sitting, standing, walking) but rather 2 factors (basic skills, komplex skills?); reliability - some categories may be redundant (BUT - needed during use of the MOVE programme), scale analysis - hierarchical and onedimensional structure for the 16 subscales, enhances construct validity and indicates unidimensional theoretical construct (amount of independence?); TDMMT should be altered: some items should be omitted, some allocated to other levels, some ordered in a different sequence; CRITIQUE: TDMMT was not developed as a test to measure change, but as a tool to guide instruction and document progress; however, the changed order of itemy may make it necessary to administer the TDMMT with more care, because it cannot be assumed that items are ordered by difficulty	journal article	English	Netherlands	main topic
103	van der Putten, Annette A. J.	2005	History/Development; Study; Study reporting	Dissertation with several studies on the effects of the MOVE programme	dissertation	English	Netherlands	main topic
104	van der Putten, Annette A. J.	2006	Assessment; Study; Derivatives	psychometric analysis of the TDMMT; change in item sequence, part of the dissertation	journal article	Dutch	Netherlands	main topic
105	van der Putten, Annette A. J.	2007	Study	Summary of the dissertation	journal article	Dutch	Netherlands	main topic
106	van der Putten, Annette A. J.	2008	Study	Summary of the dissertation	journal article	German	Netherlands	main topic
107	van der Putten, Annette A. J.; Homeijer, Nicole; Vlaskamp, Carla	2006	Study	Summary of the dissertation	journal article	Dutch	Netherlands	main topic
108	van der Putten, Annette A. J.; Reynders, Koop; Vlaskamp, Carla; Nakken, Han	2004	Study; Implementation	Goals in MOVE should be aimed at independent living skills like communication, play, interaction etc. Analysis of the goals in the MOVE programme in six centers for special education. Concrete skills 96%, selfcare 14%, mobility 54%, social functioning 23%; childs interest 52%. DISCUSSION - focus on mobility (due to focus of the MOVE prgramme?), few on self-care (care efficiency may be hidden goal); transfer from mobility skills to functional life skills unclear, not provided by authors; Theory and implementation strategy, including training, should guide correct formulation of the goals.	journal article	English	Netherlands	main topic
109	van der Putten, Annette A. J.; Vlaskamp, Carla	2007	Study	independence in movement skills as result of MOVE activities? Quasi experimental pre-posttest with control group, 12 month interval with altered TDMMT, significant improvement in experimental group, medium effect size	journal article	Dutch	Netherlands	main topic
110	van der Putten, Annette A. J.; Vlaskamp, Carla; Reynders, Koop; Nakken, Han	2005	Study	Summary of the dissertation	journal article	English	Netherlands	main topic
111	van der Putten, Annette A. J.; Reynders, Koop; Vlaskamp, Carla; Reynders, Koop; Nakken, Han	2005	Study	independence in movement skills as result of MOVE activities? Quasi experimental pre-posttest with control group, 12 month interval with altered TDMMT, significant improvement in experimental group, medium effect size	unpublished manuscript	English	Netherlands	main topic
112	Van keer, Ines; Maes, Bea	2018	Study reporting	review of contextual factors influencing the development of children with severe to profound intellectual disabilities; reviews the study of Barnes and Whinnery (2002) briefly. not further mentioned in the discussion	journal article	English	Netherlands	one of several topics
113	Whinnery, Keith W.; Whinnery, Stacie B.	2002	Concept; Case reports	Interview with a mother about home implementation of MOVE; personal experiences and perspectives; description of the MOVE concept	journal article	English	USA	main topic

114	Whinnery, Stacie B.; Whinnery, Keith W.	2004	History/Development; Case reports	description of the MOVE development and program, primary and secondary benefits: community access and cost savings; health benefits; increased social interactions; MOVE for adults; with illustrative case reports (success stories); research on the effectiveness has begun	periodical	English	USA	main topic
115	Whinnery, Keith W.; Whinnery, Stacie B.	2007	Theory; Study reporting; Case reports	shift from developmental to functional programming for early motor intervention; describes MOVE as one possible program; six steps with examples; reports research on effectivity, further studies needed	journal article	English	USA	main topic
116	Whinnery, Stacie B.; Whinnery, Keith W.	2011	Study	Study - effects of MOVE on standing and walking in adults; trend to functional, active programming for adults; describes MOVE for adults; request for MOVE for adults; study with 5 adults - independent variable MOVE, dependent variable standing with weightbearing, walking with weightbearing; participants made improvements in standing and walking, which transferred into activities, reduced necessary support and in one case also bone density.	journal article	English	USA	main topic
117	Whinnery, Stacie B.; Whinnery, Keith W.	2012	Study	case study of one adult with severe multiple disabilities, one of the cases from 2011 study in detail; Francie gained standing and walking skills, lost weight, stopped challenging behaviours	journal article	English	USA	main topic
118	Barrell, Anneliese	2007	Brief mentions	interpretation of assessments for intervention planning; not MOVE specific	book chapter	English	UK	brief mention
119	Brach, Michael	1997	Derivatives	describes the MOTA [Mobility Test for Elderly], a derivate of the TDMMT	journal article	German	Germany	one of several topics
120	Bundy, Anita; Hemsley, Browyn; Brentnall, Jennie; Marshall, Elfreda	2012	Brief mentions	mentions the study by Low (2007) in a literature review	internet article	English	Australia	brief mention
121	Cavanaugh, Lauren K.	2005	Brief mentions	Comprehensive description of intellectual disabilities and intervention/teaching approaches relevant	book chapter	English	USA	brief mention
122	Chen, Deborah	1995	Brief mentions	very short description of MOVE as a treatment for children with hypertonia;	book chapter	English	USA	brief mention
123	Cook, Ruth E.; Klein, M. Diane; Chen, Deborah	2013	Brief mentions	Interventions should move from developmental to functional goals and approaches; MOVE as an example for a functional approach; task-analysis instead of developmental goals; teaching while the child participates in activities in natural settings; limited but positive support by studies by Whinnery&Whinnery.	book	English	USA	brief mention
124	Corbeil, Thania	2016	Brief mentions	Ethnographic study on the educability of pupils with multiple disabilities. MOVE as a possibility to enhance autonomy for children with multiple disabilities, mentions van der Putten study. Teachers play a role in motor development of children with multiple disabilities.	dissertation	French	France	brief mention
125	Gartmann, Judith; Jungmann, Tanja	2021	Brief mentions	Promoting motor skills in 3-6 year old children	book	German	Germany	brief mention
126	Gosselin, Dora Jane	2021	Brief mentions	only short mention of TDMMT, not relevant in the study	dissertation	English	New Zealand	brief mention
127	Hrčová, Jana	2018	Brief mentions	short description of the programme	dissertation	Slovanian	Slovania	brief mention
128	Hrčová, Jana	2016	Brief mentions	short description of the programme	journal article	Slovanian	Slovania	brief mention
129	Kamenopoulou, Leda	2023	Brief mentions	The book conveys the results of van der Putten's study but does not elaborate on them further.	book	English	UK	brief mention
130	Levitt, Sophie; Addison, Anne	2019	Brief mentions	very detailed overview over treatment approaches; description of MOVE; "structured for teachers and carers. Therapists will have a greater variety of methods …" MOVE is critizised for not taking adequate account of the development of deformities. "MOVE relates only to gaining function"; promotes an eclectic approach; disadvantage of focussing on standing and walking like in MOVE: "omission or inadequate training of rising from lying to standing and managing bed mobility, components of which develop in the supine and prone developmental channels. This is likely to result in an individual remaining dependent on others to be 'stood up' for walking with and without walkers, and dependency in getting out of bed or for turning in bed." Suggests parallel developmental sequences and biomechanics as a foundation for therapy	book	English	UK	brief mention
131	Long, Toby M.; Brady, Rachel	2017	Brief mentions	mentions MOVE briefly as one concept to teach basic movement skills	book chapter	English	USA	brief mention
132	McQuin, Robin U.	2005	Brief mentions	mentions MOVE very briefly	dissertation	English	USA	brief mention

133	Mensch, Sonja M.; Rameckers, E. A. A.; Echteld, Michael A.; Penning, C.; Evenhuis, Heleen M.	2015	Brief mentions	new version of the 2005 article; systematic review of psychometric characteristics of instruments on motor abilities of children with complex disabilities; eight measures found; detailled description of the properties; TDMMT was the only assessment developed for target group, with uncertain evidence on reliability and positive construct validity; interesting candidate for the purpose, but properties should be studied: test-retest (possible??), responsiveness; ideal and validated tests are lacking, but several show potential	journal article	Dutch	Netherlands	brief mention
134	Morton, R. E.; Billings, K.; Hankinson, J.; Hart, D.; Nicholson, J.; Rowlands, A.; Saunders, R.; Walter, A.	2003	Brief mentions	No data on MOVE to extract; only include for recording of locations and reports of MOVE use	journal article	English	UK	brief mention
135	Nakken, Han	2005	Brief mentions	Description of an intervention in South Africa, implementation of Conductive Education. MOVE as one possible intervention, but CE was chosen.	internet article	Dutch	Netherlands	brief mention
136	Nalbant, Sibel	2011	Brief mentions	only mentions the TDMMT once	dissertation	Turkish	Turkey	brief mention
137	Niyisabwa, Odette	2016	Brief mentions	reports about the Barnes and Whinnery study, (wrongly?) assigning it as o&m traning for children with severe vision impairments	dissertation	English	Uganda	brief mention
138	Orr, Robert	2003	Brief mentions	mobility training out of the perspective of a child with complex needs<br>Very brief mention of MOVE<br>"Passivity is the curse of special education."	book	English	UK	brief mention
139	Paleg, Ginny	2002	Brief mentions	ask the therapist - parents look for a light alternative for a wheelchair for their son; therapist: view on seating has changed from maximum support and alignment to varieties of seating and posture options with ability to learn motor skills; reasons against sitting in a wheelchair and possibilities for getting	periodical	English	USA	brief mention
140	Pratt, Brenda; Peterson, Melissa L.	2015	Brief mentions	briefly mentions MOVE as Curriculum to assess and teach basic, functional motor skills needed for adult life;	book chapter	English	USA	brief mention
141	Pyo, Yun H.	2011	Brief mentions	mentions Whinnery study	journal article	Korean	Korean	brief mention
142	Şanli, Buse Büşra	2020	Brief mentions	reports the TDMMT as one assessment of motor function	master thesis	Turkish	Turkey	brief mention
143	Schack, Thomas; Pollmann, Dietmar	2020	Brief mentions	dynamic testing: assessing potential for change; zone of proximal development (cf. Whinnery); dynamic testing in different fields; motor skills in children with disabilities: MOVE - moving from a test-oriented perspective to a combination of assessment and instruction or testing and teaching	journal article	German	Germany	brief mention
144	Scullion, Irene	n.d.	Brief mentions	only 1% of children in Schotland are educated in special schools; two special schools for children with multiple disabilities and visual impairment in Edinborough; Oakland school - Curriculum of excellence; MOVE as part of the Curriculum/policy	internet article	English	UK	brief mention
145	Strapasson, Aline Miranda; Harnisch, Gabriela Simone; Kishimoto, Simone Thiemi	2017	Brief mentions	only mentions the TDMMT once	journal article	Portuguese	Brazil	brief mention
146	van Alphen, Helena J. M.; Waninge, Ali; Minnaert, Alexander; van der Putten, Annette A. J.	2019	Brief mentions	shortly mentions MOVE, but it seems not to be in use in the Netherlands (anymore)	journal article	English	Netherlands	brief mention
147	van Alphen, Helena J. M.; Waninge, Ali; Minnaert, Alexander; van der Putten, Annette A. J.	2023	Brief mentions	Development of an activation program.	journal article	English	Netherlands	brief mention
148	Vlaskamp, Carla	1999	Brief mentions	MOVE only mentioned as one of several approaches that evolved over the last 20 years	journal article	English	Netherlands	brief mention
149	Vlaskamp, Carla; Nakken, Han	2008	Brief mentions	interventions in institutions for people with PIMD in the Netherlands and Belgium - not much research on the effectivity - first step: overview over the used methods; practitioners were asked, MOVE was not used; other methods not validated	journal article	English	Netherlands	brief mention
150	Wang'ang'a, Anne Rose Wanjiku	2014	Brief mentions	inadequate training of teachers for students with multiple disabilities in Kenya; overview of the methods used in Kenya; MOVE described as one interevention for children with "Cerebral palsy intellectual disability" in the literature review; structured questionnaire without MOVE; MOVE not used (only mobility aids n=5/30 and walking frame n=1/30; training needs: a bit of transfer and functional skills; recommendations: Acquisition of functional skills; specialised equipment and adaptive aids;	dissertation	English	USA	brief mention
151	Zoltan, Lénárt	2019	Brief mentions	MOVE briefly mentioned in a study about improvement of upper limb motor skills	dissertation	Hungarian	Hungary	brief mention

152	Benson, Kathleen; Capone, Kristin; Duch, Kimberley, Palmer-Casey, Christine	2020	History/Development; Concept; Assessment; Implementation; Curriculum	Describes MOVE within a framework for school-based PT. Describes the 6 steps and possible implemention into the school day. States that motor learning Theory supports MOVE (Thompson). Case Study of one child GMFCS IV, one child GMFCS III, where the MOVE assessment was used.	book chapter	English	USA	main topic
153	Bidabe, D Linda	2000	History/Development; Concept	motivation to develop MOVE, history	conference abstract	English	USA	main topic
154	Bidabe, D Linda	2002	History/Development; Concept	informal description of Bidabes work	periodical	English	USA	main topic
155	Bidabe, D Linda; Barnes, Stacie B.; Whinnery, Keith W.	2001	History/Development; Concept; Assessment; Theory	classifies the MOVE program; six steps; motivation to develop MOVE; Literature and concepts that MOVE is based upon; reference to Snell and others; pilot study; Barnes study; Theory: see Barnes study;	journal article	English	USA	main topic
156	French, Jenny	1996	History/Development; Concept; Implementation	short introduction of MOVE, experiences at different MOVE sites; (TELL)	periodical	English	UK	main topic
157	French, Jenny	2000	History/Development	The need to do research on the effectivity of the MOVE programme; questions like: MOVE effectivity in comparison to other methods; transfer into quality of life; health and well-being; why does it work?; change from passive to active personality;	conference abstract	English	UK	main topic
158	Herndon, Cynthia Lang	1997	History/Development; Equipment; Assessment; Derivatives	Proposal of a physical activity program utilizing some features of the MOVE program (test, goal setting, task analysis). Application remains unclear.	master thesis	English	USA	one of several topics
159	Homeijer, Nicole	2000	History/Development; Concept; Implementation	dutch description of the MOVE concept, implementation by IPSE, research by University Groningen	journal article	English	Netherlands	main topic
160	Kingsbury, Karen	1991	History/Development; Concept; Case reports	Proliferation of MOVE in the US - courses in different states in 1991; contact to Argentina and Europe	news article	English	USA	main topic
162	Goebel, Terri; McClary, Brittany	2007	Concept; Theory; Implementation	description of MOVE approach - core movement skills for more mobility, better health, enhanced participation; shift from developmental to functional interventions; principles: functional Curriculum; natural environments; family-centered; integrated therapy; partial participation, six steps, aligns with current best practice strategies, teaching sitting skills: shift from static seating and positioning techniques to teaching sitting skills;	conference abstract	English	USA	main topic
163	Barry, Margaret J.	1996	Concept; Equipment	MOVE as one of several interventions, growing use in schools, description focussing on team, use of equipment and reducing support, "Further study is needed to provide evidence for the effectiveness of this treatment method"	journal article	English	USA	one of several topics
164	Best, Anthony	1997	Concept; Equipment	short mention	book chapter	English	USA	one of several topics
165	Capone, Kristin; Hoopes, Diana; Kiser, Deborah	2005	Concept	Description of MOVE approach and use in schools - in a book about CP	book chapter	English	USA	main topic
166	Capone, Kristin; Hoopes, Diana; Kiser, Deborah; Rolph, Beth	2007	Concept	Description of MOVE approach and use in schools - in a book about CP	book chapter	English	USA	main topic
167	Ellis, Julie	1996	Concept	MOVE does not follow developmental sequence; common definition as top-down, activity based programme; designed for children over 7 years of age with severe developmental disabilities; 1996: 41 certified MOVE Trainers in the US; "concentrates everybody´s energy in the same direction"; case reports, success stories;	periodical	English	USA	main topic
168	Fox, Mark	2004	Concept	short, classic description of MOVE and TDMMT; teaching can include Bobath or Conductive Education approaches; teachers involved in teaching skills	book	English	UK	one of several topics
169	Freivogel, Susanna	2018	Concept	mentions MOVE as an educational/learning-based approach, meaningful goals set by team, based on TDMMT, no randomized controlled studies	journal article	German	Germany	one of several topics
170	French, Jenny	1997	Concept; Assessment; Implementation	expanded version of French 1996	journal article	English	UK	main topic
171	Goldsmith, John; Goldsmith, Liz	2013	Concept	emphasis on postural alignment and care; positive if carefully applied; critique: emphasis on short term goals, disregard of the principles of postural care	book chapter	English	UK	one of several topics
172	Hart, Melanie A.; Shaughnessy, Michael F.	2006	Concept; Assessment; Curriculum	relatively general description of MOVE and the TDMMT, not much in depth information; target population: students with sensory impairments	book chapter	English	USA	one of several topics
173	Hussey, David	1997	Concept; Implementation; Curriculum	describes different curricula: the national Curriculum, the developmental Curriculum (where severely disabled children learn early skills) and the complementary Curriculum with programs like MOVE, where the pupils take part in relevant purposeful activities combined with high but realistic expectations of the child's progress;	book chapter	English	USA	one of several topics

174	Ikeda, Yoshifumi	2021	Concept; Assessment	description of the main ideas of the MOVE programme and the TDMMT;	journal article	Japanese	Japan	one of several topics
175	Imray, Peter; Hinchcliffe, Viv	2013	Concept; Curriculum	importance to maximise chilren's mobility potential; much effort done by MOVE; "create repeated opportunities for the meaningful practice of functional skills as a part of daily life"	book	English	UK	one of several topics
176	Kopriva, Peter	2018	Concept	It briefly and accurately describes the key points of the MOVE concept.	book chapter	English	USA	main topic
177	Lambert, Mike	1998	Concept	descriptive comparison of MOVE and CE; participants: problems with movement and mobility, various causes; only bar to inclusion is medical advice not to sit, stand or walk; children and adolescents, program also used for adults; research: pilot study and Elkins; program can be implemented by all caregivers, parents, siblings, ...; three traning levels: provider, mentor, international trainer; used at school, center, home, community; more usually implemented in special school settings; applied in all naturally occuring opportunities; normal furniture and special equipment: supportive chair, chair frame, forward-leaning gait trainer, stander with wheels; assessment by TDMMT and prompt reduction plans; incorporate into IEP; graduates sit, stand and walk independently within home or community or still require some assistance; additional benefits are typically reported<br>MOVE translated into German, French, Spanish, Italian and Japanese	journal article	English	UK	main topic
178	Laughlin, Michael K.	2013	Concept	not practical for the instruction for students with low incidence disabilities; only focusses on sitting, standing, walking; intensive training and expensive equiptment needed; excessive paperwork	dissertation	English	USA	one of several topics
179	Maier-Michalitsch, Nicola J.	2009	Concept	about the role of physiotherapy in schools for physically disabled children; describes MOVE concept	book	German	Germany	one of several topics
180	Miller, Freeman; Bachrach, Steven J.	2006	Concept	good and short description of the concept in the encyclopedia-part of the book (under therapy)	book	English	USA	one of several topics
181	Penn, Claudia	2020	Concept; Implementation	detailed description of the MOVE program: main ideas, possible to implement in different settings, six steps	periodical	German	Austria	main topic
182	Pruckler, Bonnie	nd	Concept; Implementation	explanation of the 4 stages of learning; case stories; examples of toddlers;	video	English	USA	main topic
183	Schack, Thomas; Guthke, Jürgen	2003	Concept; Assessment	dynamic testing: assessing potential for change; zone of proximal development (cf. Whinnery); dynamic testing in different fields; motor skills in children with disabilities: MOVE - moving from a test-oriented perspective to a combination of assessment and instruction or testing and teaching	journal article	English	Germany	one of several topics
184	Schack, Thomas; Pollmann, Dietmar	2014	Concept; Assessment	MOVE as compensatory intervention to enhance motor development in children and adolescents with disabilities	book chapter	German	Germany	one of several topics
185	Schomerus, Riclef; Penn, Claudia	2023	Concept	motor training needs many repititions, only possible with a team approach in schools for children with physical disabilities; independence makes participation possible; six steps of MOVE; situation of MOVE in Germany and Austria, charity	periodical	German	Germany	main topic
186	Shaw, Christine	2008	Concept	Teamwork: essential role in MOVE	journal article	English	UK	main topic
187	Toyama, Tomatiro Akiyama	2003	Concept	describes different approaches for CP diagnostik and therapy; "total rehabilitation" including medical and social approaches over the lifespan; MOVE as one approach for rehabilitation of school children; six steps, age appropriate goals, easy to understand, reduces caregiver burden	journal article	Japanese	Japan	one of several topics
188	Vermeer, Adri; Tamboer, T.; Groenhuijzen, G.	2005	Concept; Assessment	informs about the same institution in south africa as Koop Reynders, where MOVE was thought to be one suitable approach for the clientele, but CE was finally chosen; mentions TDMMT briefly as one possible assessment	book chapter	Dutch	Netherlands	one of several topics
189	Williams, Lowri; Wharton, Leigh	2020	Concept; Implementation	implementation of MOVE at one school in Wales; concept and progress	periodical	English	UK	main topic
190	Penn, Claudia	2020	Implementation	the role of school assistants in the MOVE program, spend most time with the child, equal members of the team;	periodical	German	Austria	main topic
191	Ringer-Neumann, Elisabeth	2020	Implementation	experiences of a physiotherapist with MOVE: often inactivity in children and missing knowledge in educational staff and missing interdisciplinary cooperation. MOVE gives framework to work on common goals and activate the children during school days.	periodical	German	Austria	main topic
192	Ammann-Reiffer, Corinne; Bastiaenen, Caroline H. G.; Bie, Rob A. de; van Hedel, Hubertus J. A.	2014	Assessment	discusses reliability, measurement error and responsiveness of measures of gait function (modified COSMIN)<br>TDMMT: reliability (van der Putten et al. 2005, Tedla et al. 2009) COSMIN poor, modified COSMIN fair.	journal article	English	Switzerland	one of several topics

193	Bidabe, D Linda	2009	Equipment; Assessment; Derivatives	description of the Hygiene and Toileting program; goal, six steps (a bit different to the original MOVE steps), Top Down Toileting Assessment with Hand and Arm use and Communication	book	English	USA	main topic
194	Block, Martin; Hornbaker, Jillian L.; Klavina, Ajia	2006	Assessment	Presents the Functional assessment of students with severe disabilities (FASSD), some sections and items similar to the TDMMT.	journal article	English	USA	one of several topics
195	Burton, Allen W.; Miller, Daryl E.	1998	Assessment	detailed review of the TDMMT;<br>"Mobility Opportunities Via Education is a top-down, activity based Curriculum designed to teach infants, preschoolers, school-age children, adolescents, and even young adults basic, functional movement skills needed for adult life in home and community environments"<br>Purpose of the TDMMT: instructional planning and evaluation of progress;<br>TDMMT: criterion-referenced instrument composed of 74 skills organized into 16 sitting, standing, and walking skill headings; 4 grad levels; administered retrospectively by parent, caregiver, or teacher; entry skill marked, lower skills assumed to be achieved; no summary or composite scores;<br>no reliability information; reliability difficult to establish due to TDMMT properties (retrospective nature), reliability between caregivers and teachers like in PEDI might be apporpriate; Intrarater reliability almost impossible to examine; test-retest reliability could be tested for each of the 16 categories;<br>Validity: no information available; success of MOVE related to construct validity; Content validity should be reported; experts could be queried about the validity of the content of the TDMMT and the three assessments.<br>effectivity: progress of previously regressing students support MOVE being more effective than no intervention at all.	book	English	USA	one of several topics
196	Dincher, Andrea; Dincher, Lena M.	2023	Assessment	reviews motor screenings, mentions the TDMMT with some psychometric properties, but not all validation studies applied	journal article	English	Germany	one of several topics
197	Dumas, Helene M.; Fragala-Pinkham, Maria A.; Moed, Richard	2021	Assessment	scoping review of assessments; ambulation with assistive devices; not really understands the use of the TDMMT ("use with rifton pacer"), not as one step of the MOVE Curriculum	journal article	English	USA	one of several topics
198	Kern County Superintendent of Schools	2014	Equipment; Assessment; Derivatives	describes MOVE toileting care programme	book	English	USA	main topic
199	Reichenbach, Christina	2017	Assessment	Description of the TDMMT and its psychometric properties	book chapter	German	Germany	one of several topics
200	Ross, Samantha M.; Case, Layne; Leung, W.	2016	Assessment	introduces a conceptual framework to support the process of defining PA, selecting appropriate PA tools for children with disabilities, and discussing research outcomes; ICF as template; traditional focus on activity and body structures and function; need for performance-oriented measures; Guidance to select appropriate measures, TDMMT as one measure of activity/participation)	journal article	English	USA	one of several topics
201	Schack, Thomas	2012	Assessment	based on 2003 article, almost the same content	book chapter	English	Germany	one of several topics
202	Torkildson, Laura	2001	Assessment	Description of the TDMMT, use of the TDMMT in Minnesota and North Dakota	master thesis	English	USA	one of several topics
203	Vlaskamp, Carla	2008	Assessment	TDMMT meets the need for functional motor assessments in PIMD, unlike assessing motor milestones; TDMMT provides information about the support needed to sit, stand and walk; need for assessments for this population	book chapter	English	Netherlands	one of several topics
204	Barnes, Stacie B.	1996	Study	Reviews different theories on the relationship of motor and cognitive development. Describes dynamic systems Theory and Vygotsky's zone of proximal development, and the role of scaffolding/structured to reach the next skill level. Structured guidance in motor learning eg. in naturalistic training (training in natural environments). MOVE as a naturalistic apporach. Proposes a single subject study to answer, if MOVE is more effective in increasing functional motor skills than traditional approaches, and if the increased motor skills translate into increased social interaction.	conference abstract	English	USA	main topic
205	Barnes, Stacie B.; Whinnery, Keith W.	1997	Theory	theoretical background (dynamical systems Theory, zone of proximal development, structured guidance, naturalistic therapy)	journal article	English	USA	main topic
206	Bühnen, Martina	2020	Theory	MOVE in the context of modern motor development theories	periodical	German	Germany	main topic
207	Schomerus, Riclef	2000	Theory	adaptational motor development as a theoretical foundation for MOVE	conference abstract	English	Germany	main topic

212	Adams, Lori	2007	Implementation	Ideas for teaching skills in everyday activities; School becomes MOVE model site	video	English	USA	main topic
213	Ashdown, Rob; Darlington, Chris	2007	Implementation; Curriculum	Describes the transition process in special education schools in a local authority. Ressource bases are established for students with PMLD, with specially trained staff. The MOVE programme has been adopted in the two schools and is described as a firmly established best practice in the OFSTED reports, and a means to promote inclusion of the pupils with PMLD.	journal article	English	UK	one of several topics
214	Bush, Anne	2000	Implementation	roles within the MOVE team	conference abstract	English	UK	main topic
215	Kern County Superintendent of Schools	1999	Implementation	practical worksheets	booklet	English	USA	main topic
216	Kern County Superintendent of Schools	2000	Implementation	instruction on basic and advanced transfers - sit to stand from chair and floor, stand to sit from chair and floor, wheelchair to chair, to and from toilet, into bathtub, …	video	English	USA	main topic
217	Gumprich, Andrea	2003	Implementation	Describes a movement and activation programme at her school, which combines MOVE elements with other tasks - an example how MOVE is implemented not in its original meaning	periodical	English	Germany	main topic
218	Haslhofer, Barbara	2020	Implementation	Implementation from the perspective of a MOVE practitioner/trainer in a child residential center in Austria; fixed routines make it manageable to implement MOVE into everyday schedule	periodical	German	Austria	main topic
219	MOVE - Bewegung fürs Leben	n.d.	Implementation	Description and items of the quality mark	grey literature	German	Austria	main topic
220	McGuire, Kanna	2000	Implementation	severely disabled students often not included into mainstream education due to "lack of understanding how to include and lack of method how to evaluate and measure their progress". Multidisciplinary approach, person first, disability secondary;<br>MOVE as a means to inclusion by narrowing the gap between severely disabled and typically developing students	conference abstract	English	USA	one of several topics
221	Praxmarer, Susanne	2020	Implementation	Start of the implementation of MOVE at one school in Austria	periodical	German	Austria	main topic
222	Rasmussen, Brigitte	2000	Implementation; Curriculum	implementation in a school in Denmark; transdisciplinary class teams; MOVE integrated into Curriculum for pupils with complex difficulties to access activities; MOVE can be used together with Conductive Education	conference abstract	English	USA	main topic
223	Schmidtke-Wasels, Barbara	2020	Implementation	implementation in a school in Germany, effects on students	periodical	German	Germany	main topic
224	Schmidtke-Wasels, Barbara	2023	Implementation	implementation in a school in Germany, effects on students	periodical	German	Germany	main topic
225	Schreiberhuber, Petra	2020	Implementation	MOVE from the perspective of a physiotherapist	periodical	German	Austria	main topic
226	Whinnery, Stacie B.	2004	Implementation	instruction on the first two steps of the MOVE program	video	English	USA	main topic
227	Schweitl-Öztürk, Barbara	2020	Implementation	MOVE in an inclusive classroom; case report of a young student with tetraparesis	periodical	German	Austria	main topic
228	Urschütz, Thomas; Bernhofer, Barbara	2020	Implementation	implementation in one school for special education, useful, childrens wish and parents needs; IEP; MOVE practitioner and trainer training; success	periodical	German	Austria	main topic
229	Kern County Superintendent of Schools	2009	Derivatives	step-by-step visualisation of four levels of toileting routines;	poster	English	USA	main topic
230	Pruckler, Bonnie	2004	Equipment	MOVE is not only about equipment, but to leave equipment behind	video	English	USA	main topic
231	Low, Sheryl A.	2011	Equipment	Investigates the use of support walkers; Increased use of support walkers, in part due to the development of the MOVE program	journal article	English	USA	one of several topics
232	Noble, Elena	2023	Equipment; Derivatives	describes teaching toilet skills with prompts	internet article	English	USA	one of several topics
233	Ott, Donna DeSanto; Effgen, Susan K.	2000	Curriculum	Very low rates of mobility and transfer behaviors in preschool classrooms. PT should focus their intervention on transfer and mobility behaviors. Understandable that schools use MOVE or CE to increase active movements.	journal article	English	USA	one of several topics
234	Ministry of Education and Science of the Russian Federation (Ministerstvo obrazovaniya i nauki Rossiyskoy Federatsii)	2016	Curriculum	lists MOVE as one of several recommended educational programs for use with students with disabilities, depending on the characteristics of the students and the educational goals as formulated in the SIRP (IEP)	grey literature	Russian	Russia	one of several topics
235	Blândul, Valentin C.	2010	Curriculum	Adapting mainstream curricula to include disabled students in Romania; MOVE as one possibility for students with physical disabilites	journal article	Romanian	Romania	one of several topics
236	Walker, Peter M.; Carson, Karyn L.; Jarvis, Jane M.; McMillan, Julie M.; Noble, Anna G.; Armstrong, David J.; Bissaker, Kerry A.; Palmer, Carolyn D.	2018	Curriculum	How do Educators of Students With Disabilities in Specialist Settings Understand and Apply the Australian Curriculum Framework?	journal article	English	Australia	one of several topics

237	Brach, Michael	1997	Brief mentions	Decribes in a general way the movement oriented work in a home for elderly people; MOTA as a TDMMT derivate is shortly mentioned, but the approach does not follow the MOVE philosophy	journal article	German	Germany	brief mention
238	Brach, Michael	2010	Derivatives	describes the MOPTA (Mobility Test for Patients in Acute Care), used in evaluation of interventions in elderly; based on the MOTA, based on the TDMMT; 17 items, amount of assistance is measured: Without assistance, device assistance, personal assistance, complete takeover; interestingly: in the TDMMT, device assistance counts as more assistance than personal assistance (because of weight support);	post doctoral thesis	German	Germany	one of several topics
239	Brach, Michael; Hasenritter, J.; Kirchner, E.; Bauder-Mißbach, H.; Betschon, E.; Eisenschink, A. M.; Drabner, A.; Panfli, E.	2012	Derivatives	MOTPA test for patients in acute care; includes mobility in lying position, validation, study protocol, additional file (test and manual) not retrievable	protocol	English	Germany	one of several topics
240	Brach, Michael; Jekosch, Sabine; Schulz, Henry; Dierbach, Oskar; Heck, Hermann	1995	Derivatives	describes the adaptation of the TDMMT for old people. New stages (independent, use of equipment support, person support, no success); validation results very high objectivity and reliability; can be used for assessment, control and dokumentation of movement oriented work with very old people; MOVE should be adapted for the use with this population	conference abstract	German	Germany	one of several topics
241	Brach, Michael; Jekosch, Sabine; Heck, Hermann	2000	Derivatives	describes the development of the MOTA, an adaptation of the TDMMT for very old people and it's validation results. Suggests that the MOVE Curriculum should be adapted for the use with very old people.	conference abstract	English	Germany	one of several topics
242	Brach, Michael; Wissemann, A.; Schulz, Henry; Dierbach, Oskar; Heck, Hermann	1997	Derivatives	Describes the pilot use of the TDMMT for very old people and possible adaptations. Some items may be omitted, some added. Sitting on the floor is not feasible, MOVE equipment was not used	conference abstract	German	Germany	one of several topics
243	Jekosch, Sabine; Brach, Michael; Schulz, Henry; Dierbach, Oskar; Heck, Hermann	1998	Derivatives	describes the MOTA and it's validation results	conference abstract	German	Germany	one of several topics
244	Kern County Superintendent of Schools	2009	Equipment; Case reports; Derivatives	assessment profile used for the MOVE for adults program	booklet	English	USA	main topic
245	Kern County Superintendent of Schools	2009	Derivatives	worksheets used in the Hygiene and Toileting program	booklet	English	USA	main topic
246	Mensch, Sonja M.; Möhlen-Tonino, Marijke von der	2006	Derivatives	Case description of a girl GMFCS V. No suitable assessment available, including TDMMT (too big steps, too low sensibility); development of the MOVAKIC for developmental levels from 0 to 12 months.	journal article	English	Netherlands	one of several topics
247	Wallace, Nadine; Burgwin, Kathryn; Burton, Jessica	2016	Derivatives	Implementation of the MOVE Hygiene and Toileting program in one school in western PA, using mobility skills for toileting routines; nonscientific data collection shows trend to increased use of standing and sitting toileting routines and less lying routines	internet article	English	USA	main topic
248	Young, Beverly	2008	Derivatives	describes how adults with disabilities could go horseback riding with the "independence saddle" which was based on an Australian saddle; saddle with backrest and forearm support; describes improvements in several domains	periodical	English	USA	one of several topics
